# Genome-wide association study reveals putative genetic determinants of longitudinal immune responses to avian influenza and Newcastle disease in chickens

**DOI:** 10.1186/s12711-026-01077-2

**Published:** 2026-08-11

**Authors:** Haile Berihulay, Wei Luo, Ainong Lao, Lin Chuxiao, Endashaw Jebessa, Xian Zou, Jian Ji, Hao Qu, Peng Chen, Manshan Cai, Dingming Shu, Chenglong Luo

**Affiliations:** 1 State Key Laboratory of Swine and Poultry Breeding Industry, Guangdong Provincial Key Laboratory of Animal Breeding and Nutrition, Institute of Animal Science, Guangdong Academy of Agricultural Sciences, Guangzhou, 510640 China; 2 Guangdong Laboratory for Lingnan Modern Agriculture, Guangzhou, 510642 China; 3 Department of Biotechnology, College of Natural and Computational Science, Aksum University, 1010 Aksum, Tigray Ethiopia

## Abstract

**Background:**

Avian Influenza virus (AIV) and Newcastle disease virus (NDV) are highly contagious immunosuppressive pathogens that cause high mortality rates and economic losses in the global poultry industry. Although vaccination to enhance the host antibody response remains the main control strategy, the genetic basis of this variation is poorly understood. In this study, we used a genome-wide association study (GWAS) to investigate the genetic architecture of the longitudinal antibody responses to AIV (subtypes H5, H7, and H9) and NDV in chickens.

**Results:**

To identify the genomic regions and candidate genes associated with the longitudinal antibody response to AIV and NDV after immunization, we conducted a GWAS using 1,359,927 SNP markers in an advanced intercross line (AIL) of the F19 resource population. We found moderate heritability for both the traits. Based on these associations, we identified the genomic regions and candidate genes for both traits. Association analysis revealed two significant SNPs located on chromosome 3, rs316474025 near NFKBIE and SLC35B2 [rs316474025], and rs312870707 near ATG5 gene, that are associated with viral antibody titers against AIV subtypes H5 and H7, respectively. Additionally, one variant on chromosome 27, rs318005864 near DDK12 and RPL19 genes, was strongly linked to the antibody response associated with NDV at mid-phase (40 days post-immunization). Furthermore, another variant, rs737164663, near FRMD5 and GLCE genes, was strongly linked to the antibody titer associated with both AIV subtype H9 and NDV in the late phase (60 days post-immunization). Genes located in these regions may be responsible for the immune response in chickens. Before implementation in breeding programs, the efficacy and long-term persistence of selecting these antibody response traits should be validated in larger independent cohorts.

**Conclusions:**

We confirmed the presence of genetic variability and identified SNPs significantly associated with antibody titer traits in F19 chickens. These findings highlight genomic regions contributing to variation in antibody responses and provide valuable information for improving antibody-related traits through selective breeding programs.

**Supplementary Information:**

The online version contains supplementary material available at 10.1186/s12711-026-01077-2.

## Background

Poultry farming plays a vital role in global food security by providing a major source of high-quality animal protein and income for communities worldwide [1]. Poultry production is widely practiced due to its cost-effectiveness, short production cycles, and efficient utilization of agricultural resources [2]. Among poultry species, chickens (*Gallus gallus domesticus*) are one of the most valuable livestock resources, contributing to both intensive commercial production systems and extensive smallholder operations in rural and urban settings [3, 4]. In addition to their economic importance, chickens also serve as important model organisms for studies in avian biology and immunogenetics [5]. Infectious diseases remain a major challenge for poultry production and public health, particularly those caused by zoonotic pathogens. Avian influenza virus (AIV) and Newcastle disease virus (NDV) are among the most economically significant viral pathogens affecting poultry worldwide [5]. Chickens play a central role in the epidemiology of both viruses, acting as major reservoirs and amplification hosts that sustain viral circulation within poultry populations. They also underpin vaccination-based control programs aimed at limiting viral spread and reducing production losses [6]. The outbreaks of AIV and NDV can result in severe economic consequences, including high mortality in susceptible flocks, trade restrictions, and disruptions to food supply chains [7].

Commercial poultry systems incur substantial costs associated with disease prevention, including vaccination programs and biosecurity measures. Improving vaccine-induced immune responses in chickens is therefore essential for enhancing flock health, productivity, and sustainability [8]. One promising approach is indirect genetic selection based on immune response traits, which can complement conventional vaccination strategies by improving the magnitude and persistence of host immune responses [9]. Immune responsiveness is a heritable and economically relevant trait in poultry and plays a key role in determining variability in antibody production following vaccination [10, 11].

Genetic improvement of immune responsiveness offers a sustainable strategy to enhance vaccine efficacy and immune resilience in poultry populations [11]. However, selection for immune-related traits remains challenging because immune competence is difficult to measure directly and consistently under commercial conditions [9].

Consequently, antibody titers have been widely used as quantitative indicators of humoral immune response, reflecting individual variation in the ability to mount and maintain vaccine-induced immunity [12]. Substantial variation in antibody responses has been reported among chicken lines, populations, developmental stages, and antigenic challenges [9, 12].

Understanding the temporal dynamics of antibody responses to NDV and AIV vaccination is critical for improving immunization strategies and informing genetic improvement programs; integrating longitudinal antibody measurements with genomic data provides a powerful framework to dissect the genetic architecture of immune response dynamics [6, 13]. Advances in genomic technologies, particularly the historical development of genome-wide association studies (GWAS), have facilitated the identification of genetic variants and genomic regions linked to complex traits, including immune responsiveness and vaccine-induced antibody production [14]. GWAS has been successfully applied in livestock to investigate polygenic traits related to immune function; for example, resilience-associated immune related traits have been reported in pigs [15]. In chickens, Luo et al. [16] conducted a GWAS in an F2 population and identified several SNPs associated with antibody responses to NDV vaccination. Similarly, Liu et al.[14], performed GWAS and genomic selection analyses of antibody responses to AIV and NDV at early developmental stages. However, these studies focused on single or early time points and did not evaluate immune responses across multiple post-vaccination stages, limiting insights into immune persistence. Despite growing interest in immune-related GWAS in livestock [4, 17, 18], studies addressing the genetic architecture of longitudinal vaccine-induced immune responses in chickens remain limited.

Advanced intercross populations provide a powerful resource for dissecting the genetic basis of complex traits due to increased recombination and enhanced mapping resolution. The F19 advanced intercross line (AIL) used in the present study represents a genetically diverse population derived from a cross between the slow-growing Chinese indigenous Huiyang Beard chicken and a fast-growing commercial broiler (Line A), followed by 19 generations of random intercrossing [4]. This population exhibits high genetic diversity and substantial phenotypic variation inherited from divergent founder lines, making it well suited for high-resolution GWAS of complex traits, including vaccine-induced immune responses. Although originally developed to study growth-related traits, the genetic structure of the F19 AIL is also highly suitable for mapping immune response phenotypes [4].

Therefore, in this study, we used 572 individuals from the F19 AIL population to conduct a GWAS of antibody responses to AIV and NDV vaccination measured at four time points. The objectives were to identify genetic variants, genomic regions, and candidate genes associated with the magnitude and persistence of vaccine-induced immune responses and to provide insights into the genetic mechanisms underlying immune response dynamics. These findings are expected to contribute to the development of genetic selection strategies aimed at improving immune responsiveness and vaccination efficiency in chickens.

## Materials and methods

### Ethics statement

The study was performed in strict accordance with the guidelines for experimental Animal Care Committee of the Institute of Animal Science, Guangdong Academy of Agricultural Sciences (Guangzhou, People’s Republic of China) with the approval number GAAS IAS-2009-73.

### Experimental population and Phenotypic measurements

A total of 587 individuals from the F19 resource population of advanced intercross line (AIL) chickens were used to investigate antibody response traits to NDV and AIV. The F19 AIL was derived from an initial cross between the High-Quality Chicken Line A (HQLA) and the Huiyang Bearded (HB) chicken, a Chinese indigenous breed [16]. HQLA is a specialized line developed through crossbreeding and selection between Anka Red chickens and other yellow-feathered broiler lines, characterized by rapid growth and superior meat quality.

All birds were housed individually under uniform management, hygiene, and environmental conditions, with ad libitum access to feed and water. Vaccination was conducted using a combined immunization strategy: a commercial live NDV vaccine (LaSota strain; Intervet International B.V., Boxmeer, The Netherlands) and inactivated AIV vaccines representing the H5, H7, and H9 subtypes. The NDV and AIV vaccines were administered as separate formulations rather than as a recombinant vector vaccine. All vaccines were applied via the eyedrop route at 249 days of age, following the manufacturers’ instructions.

Blood samples were collected at four time points: one day prior to immunization (baseline; P0) and at 20 (P1), 40 (P2), and 60 (P3) days post-immunization. Collected blood samples were allowed to clot overnight at room temperature and subsequently centrifuged at 1000 rpm for 10 min. The resulting serum was separated and stored at − 20 °C until analysis. Antibody responses to NDV and AIV were quantified using the hemagglutination inhibition (HI) assay according to the guidelines of the World Organization for Animal Health (OIE) [19].

### Genomic sequencing, SNP calling and quality control

At 249 days of age, 2 mL of whole blood was collected from the wing vein of 587 chickens and transferred to EDTA-K2 anticoagulant tubes for analysis. The samples were gently mixed and stored at − 20 °C until further processing. Genomic DNA was extracted using the phenol–chloroform method, according to standard protocols. DNA quality and integrity were assessed using agarose gel-electrophoresis and spectrophotometric analysis. Only samples meeting the following criteria were retained for sequencing: an A260/280 ratio between 1.8 and 2.0, an A260/230 ratio > 2.0, and a minimum DNA concentration of 50 ng/µL.

Whole-genome resequencing was performed using the DNBSEQ-T7 platform, generating 150-bp paired-end reads. After quality control, each sample yielded approximately 12 Gb of high-quality clean data, corresponding to an average sequencing depth of approximately 10 × . Read pairs were removed if either read met any of the following criteria: presence of adaptor sequences, more than 50% low-quality bases, or more than 5% ambiguous (N) bases.

Clean reads were aligned to the chicken reference genome (GRCg7a) using the Burrows–Wheeler Aligner (BWA) v0.7.17-r1188 [20]. SAMtools v1.3.1 [21] was used to sort the alignments and remove duplicate reads. Base quality score recalibration and local realignment were subsequently performed using GATK v3.8 [22], to improve mapping accuracy. Single nucleotide polymorphisms (SNPs) were identified using the BaseVar–STITCH pipeline [23]. BCFtools were applied to identify and remove individuals with potential duplication or high genetic relatedness; accordingly, 14 individuals with high genetic correlations and one individual lacking phenotypic records were excluded.

SNPs located on the sex chromosomes (Chr-Z and Chr-W) were reassigned to chromosome 40 prior to the genome-wide association analysis. Following SNP detection, genotype data were obtained for 587 individuals across 10,029,369 SNPs. Quality control was conducted using PLINK v1.9 [24], retaining samples with a call rate > 97% and filtering SNPs based on the following criteria: SNP call rate ≥ 95%, minor allele frequency (MAF) ≥ 0.05, Hardy–Weinberg equilibrium (HWE) *P* ≥ 1 × 10^−6^, and retention of SNPs on autosomes 1–38. Linkage disequilibrium (LD) pruning was performed using a sliding window of 50 SNPs, a step size of five SNPs, and an r^2^ threshold of 0.5, resulting in 1,359,927 independent SNPs for downstream analysis. After all quality control procedures, 572 individuals remained for the subsequent GWAS.

### Estimates of heritability and genetic correlation

Descriptive statistics for both traits and phenotypic correlations were performed using GraphPad Prism 5 (http://www.graphpad.com/), with *P*-values < 0.05 considered statistically significant. Genetic correlations were estimated using a restricted maximum likelihood (REML) algorithm implemented in GCTA v1.94 [25]. The total phenotypic variance (${\mathrm{V}}_{\mathrm{P}}$) was partitioned into genetic variance (${\mathrm{V}}_{\mathrm{G}}$) and residual variance (${\mathrm{V}}_{\mathrm{e}}$) as:

1 $${V}_{P}={V}_{G}+{V}_{e},$$

The heritability (${\mathrm{h}}^{2}$) of antibody responses to NDV and AIV at P0-P3 was calculated as the proportion of total phenotypic variance explained by genetic variance:

2 $$ h^{2} = \frac{{V_{G} }}{{V_{G} + V_{e} }}, $$

Finally, phenotypes that were significantly skewed antibody tires were log- to normalize their distributions. The absence of normal distribution and homogenous distribution of the within-group variances was confirmed using the Shapiro–Wilk test.

### Genome-wide association study

A genome-wide association study (GWAS) was performed to identify single nucleotide polymorphisms (SNPs) associated with antibody response traits using a mixed linear model (MLM) implemented in GCTA v1.94 [25]. The genomic relationship matrix (**G**) was constructed from genome-wide SNP markers and used to capture additive polygenic effects, thereby enabling an accurate estimation of genetic merit and reducing confounding due to population structure. For the GWAS, each SNP was fitted individually as a fixed effect in the following model:

3 $$\mathbf{y}=\mathbf{X}\mathbf{b}+\mathbf{w}\alpha +\mathbf{Z}\mathbf{u}+\mathbf{e},$$

where **y** is the vector of antibody response phenotypes; **X** is the incidence matrix for fixed effects, including sex, immunization stage (P0–P3), and the top three principal components derived from principal component analysis (PCA); **b** is the vector of fixed-effect solutions; **w** is the vector of genotypes for the SNP being tested (coded as 0, 1, or 2); and *α * is the allele substitution effect of that SNP. **Z** is the incidence matrix relating observations to random additive genetic effects; **u** is the vector of random additive genetic effects, assumed to follow:

4 $$\mathbf{u}\sim N\left(0,\mathbf{G}{V}_{g}\right),$$

where **G** is the genomic relationship matrix; and **e** is the residual vector, assumed to follow:

5 $$\mathbf{e}\sim N\left(0,\mathbf{I}{V}_{e}\right),$$

Manhattan and Q-Q plots of antibody response were generated using the CMplot package [26] in R v4.41 (https://cran.r-project.org/web/packages/CMplot/index.html). For the GWAS results, the significance and suggested thresholds were set as *P* < 1 × 10^–7.6^ (0.05/1,359,927) and *P* < 1 × 10^–6^ (1/1,359,927), respectively, according to a 5% Bonferroni correction.

### Post GWAS analysis

All SNPs that reached genome-wide or suggestive significance in the GWAS were mapped to the chicken reference genome (*Gallus gallus*, Galgal7) using the Ensemble database. Functional annotation of significant variants was performed using the Ensemble Variant Effect Predictor (VEP) tool (http://www.ensembl.org/Tools/VEP). In addition, genes located within ± 50 kb upstream and downstream of each significant SNP were retrieved using the Ensemble BioMart data-mining tool (http://www.ensembl.org/biomart/martview/). As a result of this analysis, we were able to create gene lists that contained the genes near significant SNPs identified for each immune response trait along with the genes near the identified significant SNPs. Gene Ontology (GO) enrichment analysis terms with corrected *P* values < 0.05 was performed using the Database for Annotation, Visualization, and Integrated Discovery (DAVID) online tool [27] on candidate genes identified by GWAS. The linkage disequilibrium (LD) block structure surrounding significant loci was evaluated using Haploview v4.2 [28], enabling the delineation of genomic regions potentially underlying the observed associations.

## Results

### Description of phenotypic traits

The descriptive statistics for antibody responses against AIV subtypes (H5, H7, H9) and NDV revealed clear patterns of antibody induction and persistency following vaccination (Table 1). The average mean and standard deviation of antibody titers recorded for AIV (2.97 ± 0.21) fitted a normal distribution (*P* = 0.453, Additional file 1: Fig. S1a). For AIV H5, mean antibody titers increased from 2.62 ± 0.23 at baseline (P0; Min = 2.58, Max = 3.58, Range = 1.00) to 3.21 ± 0.22 at P1 (Min = 2.81, Max = 3.58, Range = 0.78), and remained stable at P2 (3.13 ± 0.25) and P3 (3.14 ± 0.22), indicating sustained antibody levels. Similarly, H7 titers increases from 2.52 ± 0.22 at P0 (Min = 2.00, Max = 3.32, Range = 1.32) to a peak of 3.13 ± 0.18 at P1, followed by a gradual decline at P2 (3.07 ± 0.24) and P3 (3.01 ± 0.22), suggesting partial persistence of the immune response. For H9, antibody titers increased from 2.45 ± 0.18 at P0 (Min = 2.32, Max = 3.46, Range = 1.14) to 3.24 ± 0.15 at P1, then showed a slight reduction at P2 (3.10 ± 0.23) and stabilization at P3 (3.14 ± 0.17), demonstrating moderate antibody maintenance over time.

**Table 1:** Descriptive Statistics of phenotypic data of 572 individuals

Traits	Subtype	Period	Mean	SD	Min	Max	Range	CV%
AIV	H5	P0	2.62	0.23	2.58	3.58	1.00	6.86
		P1	3.21	0.22	2.81	3.58	0.78	8.25
		P2	3.13	0.25	2.58	3.58	1.00	8.27
		P3	3.14	0.22	2.81	3.58	0.78	7.11
	H7	P0	2.52	0.22	2.00	3.32	1.32	7.42
		P1	3.13	0.18	2.00	3.58	1.58	5.47
		P2	3.07	0.24	2.00	3.58	1.58	7.94
		P3	3.01	0.22	2.32	3.58	1.26	7.21
	H9	P0	2.45	0.18	2.32	3.46	1.14	5.95
		P1	3.24	0.15	2.58	3.58	1.00	4.42
		P2	3.10	0.23	2.32	3.58	1.26	7.19
		P3	3.14	0.17	2.58	3.58	1.00	5.35
NDV	Lasota	P0	2.70	0.16	2.81	3.32	0.51	4.34
		P1	3.31	0.16	2.81	3.32	0.51	5.99
		P2	3.40	0.16	2.81	3.32	0.51	6.27
		P3	3.21	0.15	2.81	3.32	0.51	5.06

Antibody titers against NDV were observed to be relatively higher than those against AIV; however, this difference was not statistically significant. For NDV, antibody titers increased markedly following vaccination and remained relatively high over subsequent periods, indicating a persistent immune response. Consistently, the overall mean antibody titer was 3.16 ± 0.16 and conformed to a normal distribution (*P* = 0.353; Additional file 1: Fig. S1b), further supporting the stability and persistence of the NDV-specific immune response. The mean titer at baseline (P0) was 2.70 ± 0.16 (Min = 2.81, Max = 3.32, Range = 0.51), reflecting limited pre-existing immunity. Antibody titers peaked at P2 (3.40 ± 0.16) and declined slightly at P3 (3.21 ± 0.15), while maintaining a consistently narrow range and low coefficients of variation (CV = 4.34–6.27%), indicating a uniform antibody response among individuals.

**Fig. 1:**
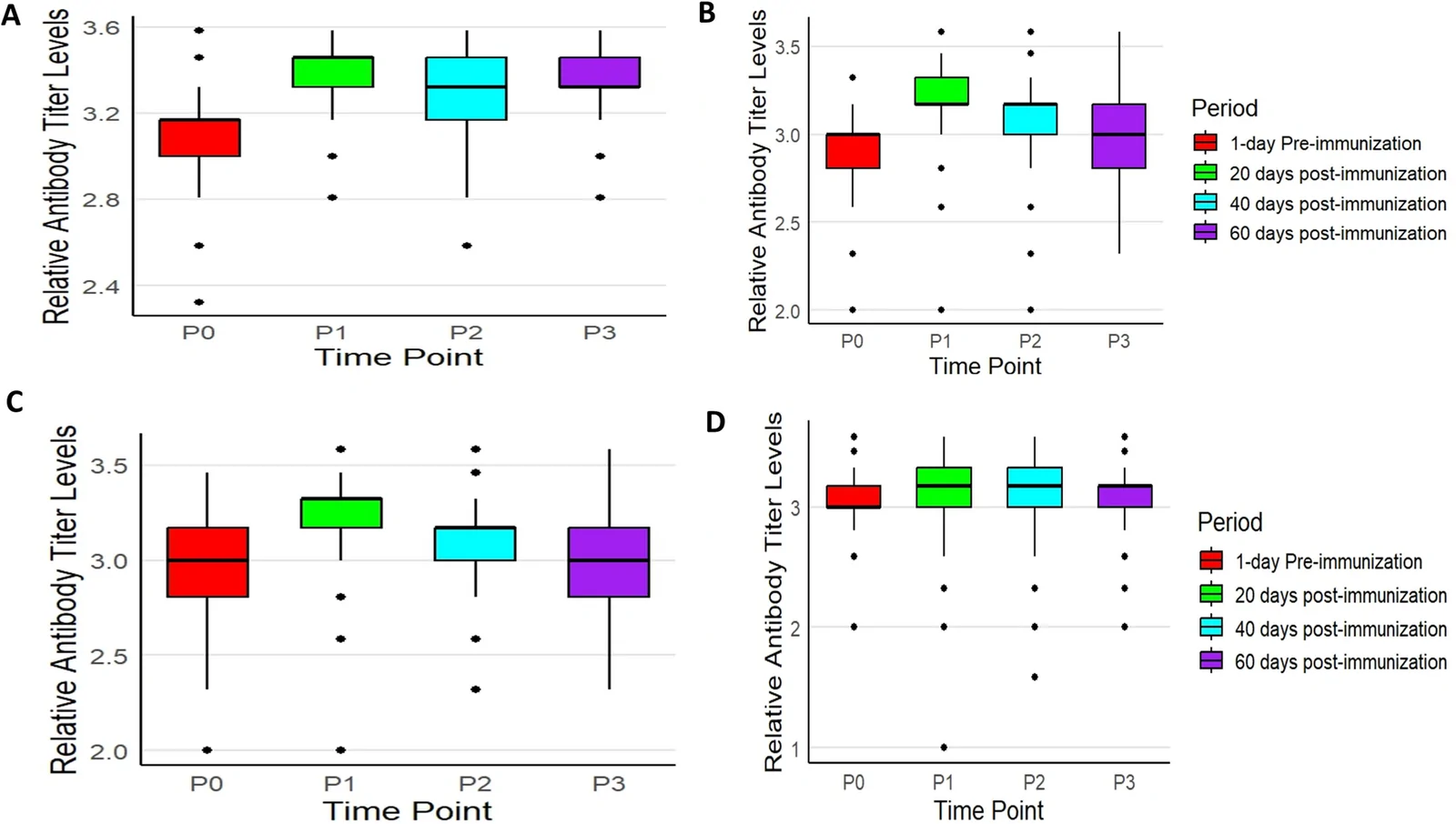
Longitudinal antibody responses (n = 431) across four-time points: P0 (1 d pre-immunization), P1 (20 days post-immunization), P2 (40 days post-immunization), and P3 (60 days post-immunization) (**a**–**d**). **a** Relationship between time point and AIV subtype H5 relative antibody titers. **b** Relationship between time point and AIV subtype H7 relative antibody titers. **c** Relationship between time points and AIV subtype H9 relative antibody titers. **d** Relationship between time points and NDV relative antibody titers

Across all subtypes, the coefficient of variation was generally highest at baseline and lowest at peak response periods, suggesting reduced inter-individual variability immediately following vaccination and moderate variation thereafter. The box-plot visualization (Fig. 1) further supports these findings, showing low median titers at P0, marked post-vaccination increases, and limited declines over time. The relatively narrow interquartile ranges at P1 indicate a homogeneous early immune response, whereas slightly wider distributions at P2 and P3 reflect moderate individual differences in antibody persistence. Overall, vaccination induced strong initial antibody responses with durable persistency, consistently detectable antibody titers across individuals, and moderate inter-individual variation for both AIV and NDV.

### Heritability and genetic correlation

Estimates of variance components, heritability, and their standard errors for antibody responses to AIV and NDV across the four sampling time points (P0–P3) are presented in Table 2. For AIV antibody responses, heritability estimates ranged from low to moderate (0.15–0.35), with the highest genetic contribution observed for the H5 subtype at P2 (0.35 ± 0.05). Similarly, antibody responses to NDV exhibited moderate heritability estimates ranging from 0.23 to 0.35, with peak heritability also detected at P2.

**Table 2:** Estimates of variance components and heritability for traits studied in AIV and NDV in 572 individuals

Traits	Subtype	Periods	Source	Vg(σ_a_^2^)	Ve(σ_e_^2^)	Vp(σ_p_^2^)	Vg/Vp(h^2^)
AIV	H5	P0	Variance	0.008	0.022	0.029	0.154
			SE	0.003	0.003	0.002	0.004
		P1	Variance	0.005	0.019	0.023	0.183
			SE	0.002	0.002	0.002	0.016
		P2	Variance	0.009	0.045	0.055	0.351
			SE	0.005	0.005	0.004	0.051
		P3	Variance	0.007	0.021	0.027	0.250
			SE	0.003	0.003	0.002	0.091
	H7	P0	Variance	0.010	0.035	0.046	0.231
			SE	0.004	0.004	0.003	0.020
		P1	Variance	0.005	0.026	0.031	0.240
			SE	0.002	0.003	0.002	0.008
		P2	Variance	0.005	0.054	0.059	0.330
			SE	0.004	0.005	0.004	0.003
		P3	Variance	0.012	0.033	0.045	0.263
			SE	0.005	0.005	0.003	0.010
	H9	P0	Variance	0.012	0.022	0.033	0.321
			SE	0.004	0.003	0.002	0.004
		P1	Variance	0.006	0.016	0.022	0.223
			SE	0.002	0.002	0.002	0.010
		P2	Variance	0.013	0.038	0.052	0.272
			SE	0.005	0.005	0.004	0.003
		P3	Variance	0.008	0.019	0.028	0.332
			SE	0.003	0.002	0.002	0.102
NDV	Lasota	P0	Variance	0.009	0.015	0.024	0.234
			SE	0.003	0.002	0.002	0.010
		P1	Variance	0.003	0.022	0.024	0.250
			SE	0.002	0.0025	0.002	0.041
		P2	Variance	0.004	0.023	0.027	0.350
			SE	0.002	0.002	0.002	0.008
		P3	Variance	0.006	0.016	0.022	0.283
			SE	0.002	0.002	0.001	0.005

AIV log transformed antibody titers against avian influenza virus (subtype H5, H7,H9); NDV log transformed antibody titers measured for Newcastle disease virus; P0 antibody titer at time point 1 d pre-challenge to immunization (log-transformed), P1 antibody titer at time point 20 d pre-challenge (baseline), P1, P2, P3 antibody titer at 20, 40 and 60 d post immunization (log-transformed);Vg(σ_a_^2^) genetic variance, Vp(σ_p_^2^) phenotypic variance, Ve(σ_e_^2^) environmental variance**,** h^2^ heritability, SE standard error

For both AIV and NDV traits, post-immunization time points P2 and P3 showed relatively moderate heritability estimates, indicating increased genetic predictability of antibody responses during these periods. These time points therefore represent optimal targets for genomic analyses and selection-related studies. Overall, NDV antibody responses displayed relatively higher average heritability (0.28) than AIV responses (0.23), suggesting a relatively higher genetic control of NDV immune responses in the F19 advanced intercross population. In general, environmental variance (V_e_) exceeded genetic variance (V_a_) across time points, indicating that non-genetic factors contributed substantially to phenotypic variation in antibody titers.

Genetic and phenotypic correlations among antibody responses to AIV and NDV across all sampling periods are summarized in Additional file 2 Tables S1 and S2. These correlations indicate the presence of shared genetic and immunological mechanisms underlying antibody responses over time. Within AIV subtypes, H5 showed a significant positive phenotypic correlation between P1 and P3 (0.35 ± 0.05; *P* < 0.05), suggesting persistent immune responsiveness across post-vaccination stages. Additionally, significant positive phenotypic correlations (*P* < 0.05) were observed between P0 and P1 for H7 and between P0 and P3 for H9.

In contrast, significant negative phenotypic correlations (*P* < 0.05) were detected between antibody titers across different AIV subtypes, such as between H7 and H9. These negative correlations likely reflect temporal dynamics and immune trade-offs, consistent with the natural decline of vaccine-induced antibody levels over time, a pattern commonly observed in poultry immune responses.

### Genome-wide association studies

**Fig. 2:**
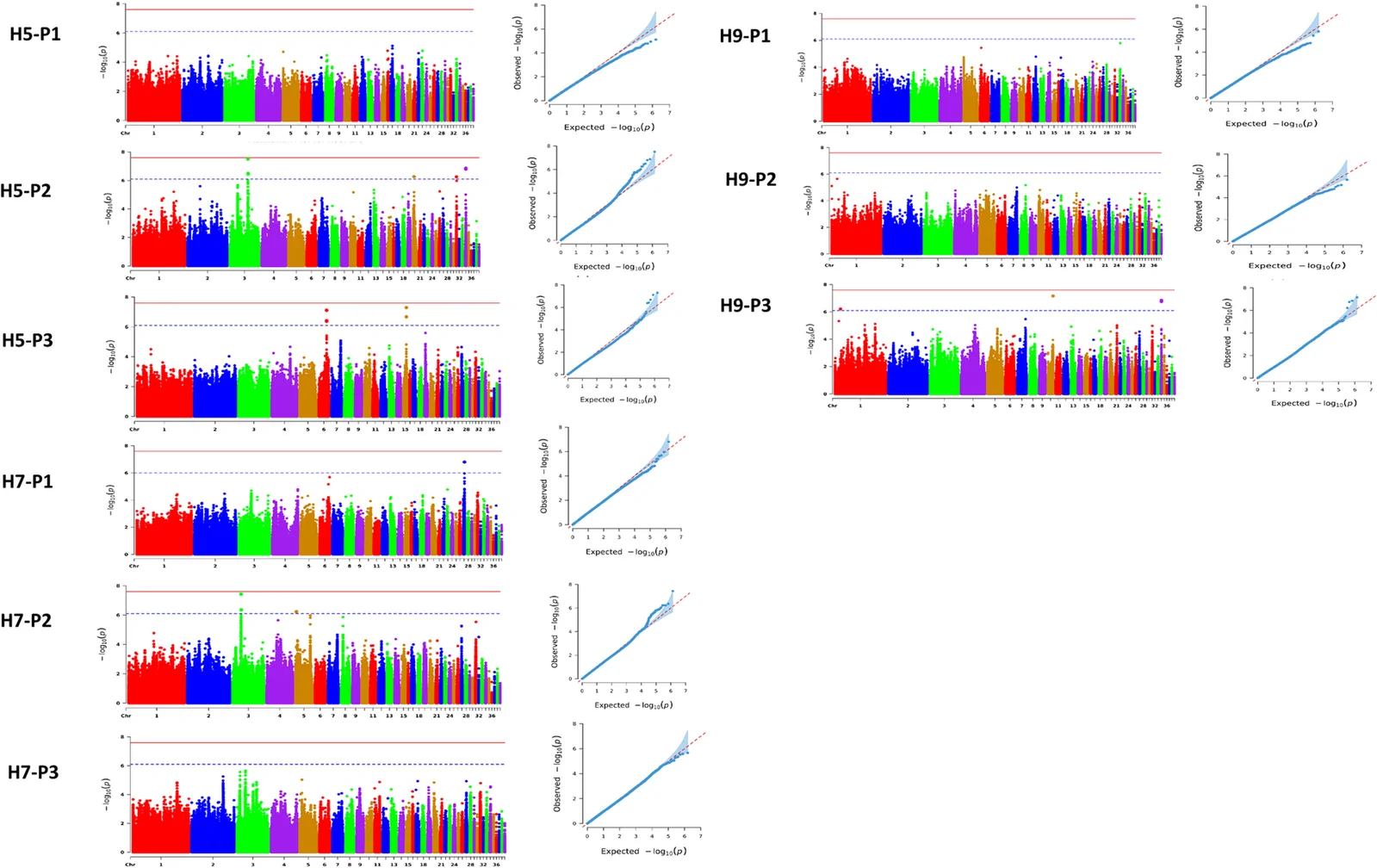
Manhattan plots for the genome-wide association analysis of AIL chickens. The genomic location (horizontal axis) is plotted against −log10(*P* value); genome-wide (*P* < 0.05) and suggestive genome-wide thresholds are shown as red and blue lines, respectively against avian influenza virus subtypes H5, H7, and H9 after postimmunization during the early, mid, and late phases (P1-P3)

**Fig. 3:**
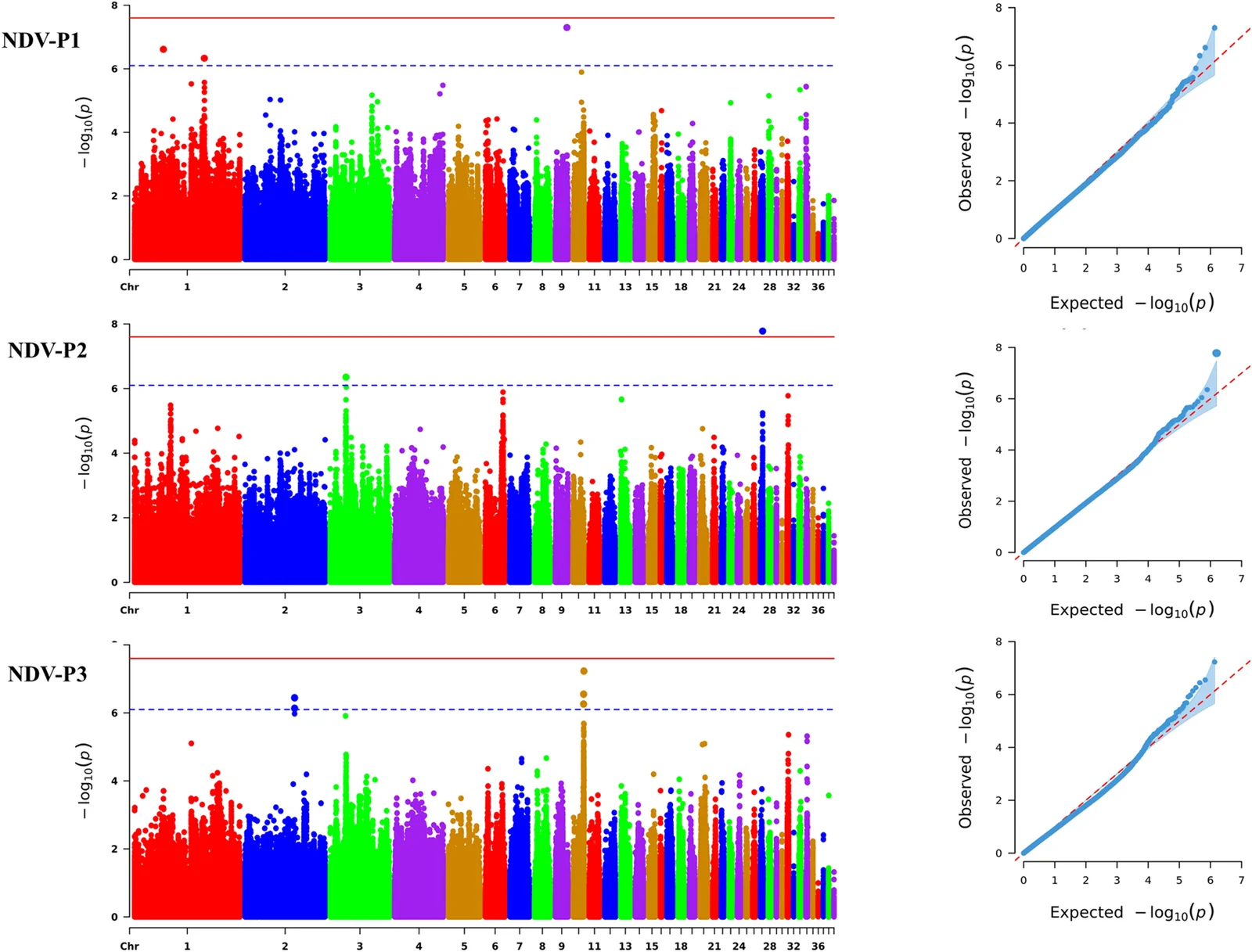
Manhattan plots for the genome-wide association analysis of AIL chickens. Genomic location (horizontal axis) is plotted against − log10(*P* value); genome-wide (*P* < 0.05) and suggestive genome-wide thresholds are shown as red and blue lines, respectively against Newcastle disease virus after post-immunization during the early, mid, and late phases (P1-P3)

Univariate GWAS of antibody response traits was performed using a mixed linear model. Multiple significant genomic regions were detected across autosomes, several of which were shared among immune response traits or were consistently identified across post-immunization time points (Figs. 2 and 3). Manhattan plots identified several SNPs exceeding suggestive and genome-wide significance thresholds, indicating loci associated with antibody titer variation, while corresponding Q-Q plots closely followed the expected diagonal, suggesting that most SNPs conform to the null hypothesis and that population stratification or test inflation was minimal. Deviations in the upper tail particularly for H5 at later time points (P2 and P3) and for NDV highlight true genetic effects on antibody responses, reflecting persistent and biologically meaningful associations. Collectively, these findings indicate that multiple genomic regions contribute to immune response and support our GWAS results, with significant SNPs likely representing genuine genetic effects rather than confounding artifacts. Furthermore, as illustrated in the P0 Manhattan and Q-Q plots (Additional file 1: Fig. S2), we performed GWAS for all traits at P0. No SNPs approached the suggestive or genome-wide significance thresholds; as expected, no biologically meaningful genome-wide significant associations were detected at this time point.

Most importantly, our GWAS dataset comprised 234 million clean reads, corresponding to 7,040.51 Gb of high-quality sequencing data from 587 individuals (Additional file 2: Table S3). Quality assessment indicated high reliability, with 97.12% of bases achieving a Q20 score and 91.46% achieving a Q30 score, supporting the accuracy of downstream genetic analyses.

Moreover, a total of 22 significant SNPs were identified, including 14 suggestive SNPs (*P* < 7.4 × 10^−7^) and six SNPs that surpassed the Bonferroni-corrected genome-wide significance threshold (*P* < 3.7 × 10^−8^). For antibody responses to AIV, 14 significant SNPs (11 suggestive and three genome-wide significant) were distributed across 10 chromosomes (GGA1, 3, 5, 6, 10, 15, 20, 27, 31, and 34; Table 3). For NDV antibody responses, eight suggestive and three genome-wide significant SNPs were detected on six chromosomes (GGA1, 2, 3, 9, 10, and 27; Table 4).

**Table 3:** Significant SNPs identified for AIV traits in 572 individuals

Trait	Subtype	SNP	Location GGA(bp)	GWAS (*p* value)	Additive effect(β)	Phenotypic variance (%)	*p*	q
AIV	H5	rs312870707^*,a^	3(67,973,638)	3.14 E−08	0.95	26	0.06^*^	0.94
		rs741794020^*a,b^	3(68,329,834)	3.16 E−07	0.89	23	0.06^*^	0.94
		rs317133482^*,b^	20(4,715,010)	5.42 E−07	0.49	21	0.19^*^	0.81
		31_1341187^*,b^	31(1,744,971)	5.55 E−07	0.73	23	0.08^*^	0.92
		rs737470279^*,a,b^	34(2,176,023)	1.36 E−07	0.62	20	0.15^*^	0.85
		rs15807865^a^	6(27,856,006)	7.71 E−07	0.37	20	0.22	0.78
		15_1282788^a^	15(10,300,045)	4.05 E−07	0.29	16	0.43	0.57
	H9	rs3555187031^*,b^	1(21,577,258)	6.09 E−07	0.70	17	0.07*	0.93
		rs737164663^*,a^	10(2,176,023)	6.91 E−08	0.73	17	0.06^*^	0.94
		rs737470279^*,a,b^	34(19,870,418)	1.40 E−07	0.52	23	0.15*	0.85
	H7	rs318005864^*,a,b^	27(4,086,707)	1.57 E−07	0.06	13	0.19*	0.80
		rs316474025^*,a^	3(29,437,437)	3.68 E−08	0.78	18	0.05^*^	0.95
		rs315514054^*,a,b^	3(29,937,549)	4.36 E−07	0.64	15	0.06^*^	0.94
		5_747704^*,b^	5(3,351,037)	5.89 E−07	0.63	14	0.06^*^	0.94

SNPs with an asterisk and highlighted in italics are significant at the genome-wide threshold, while SNPs in italics meet the suggestive genome-wide threshold. Phenotypic variance: % proportion of phenotypic variance explained by SNPs; p and q allelic frequencies, with an * marking the frequencies of the alleles corresponding to high antibody titers for avian influenza virus subtypes H5, H7, and H9. Tier classification: a (genome-wide significant and variance), b (suggestive significance and variance), and ab (significant in one metric only)

**Table 4:** Significant SNPs identified for NDV traits in 572 individuals

Trait	SNP	Location chr(bp)	GWAS (*p* value)	Additive effect (β)	Phenotypic variance (%)	*p*	q
	rs318005864^*,a^	27(4,086,707)	1.66249E−08	0.33	17	0.19*	0.80
	rs316474025^*a,b^	3(29,437,437)	4.43051 E−07	0.58	11	0.05*	0.95
	rs13653556^*b^	1(53,620,778)	2.43872 E−07	0.39	6	0.07*	0.93
	1_156858*^,b^	1(129,986,793)	4.64513 E−07	0.35	6	0.09*	0.91
	rs16695745^*a^	9(21,448,608)	5.02144 E−08	0.40	6	0.07*	0.93
	2_373300*^a,b^	2(92,404,212)	7.32367 E−07	0.59	12	0.06	0.94
	rs313229436^*a^	10(19,368,941)	5.49668 E−07	0.47	14	0.10*	0.89
	rs737164663^*a,b^	10(19,920,418)	5.89065 E−07	0.60	11	0.06*	0.94

SNPs with an asterisk and highlighted in italics SNP significant at the genome-wide threshold, while SNPs in italics SNP meet the suggestive genome-wide threshold. Phenotypic variance: % proportion of phenotypic variance explained by SNPs; p and q allelic frequencies, with an * mark the frequencies of the alleles corresponding to high antibody titers for Newcastle Disease virus. Tier classification: **a** (genome-wide significant and variance), **b** (suggestive significance and variance), and **ab** (significant in one metric only)

Notably, SNPs rs312870707 (GGA3:68,023,638 bp), rs316474025 (GGA3:29,437,437 bp), and rs737164663 (GGA10:19,870,418 bp) surpassed the genome-wide significance threshold (*P* ≤ 3.7 × 10^–8^) and were significantly associated with antibody responses to AIV subtypes H5, H7, and H9, respectively, at post-immunization time points P2 and P3 (*P* < 3.14 × 10^–8^ for H5; *P* < 3.68 × 10^–8^ for H7; *P* < 6.91 × 10^–8^ for H9).

**Fig. 4:**
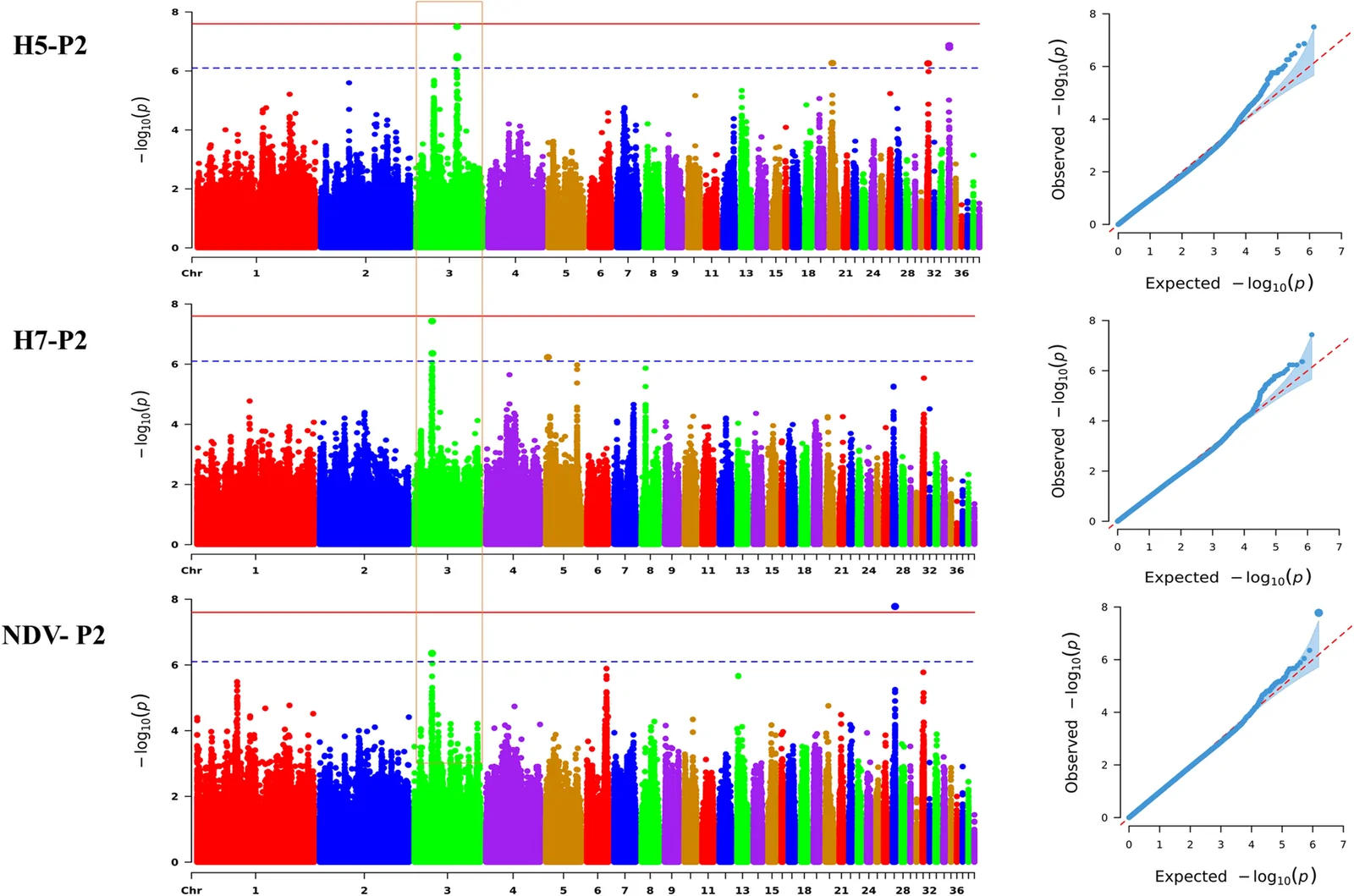
Manhattan and Q-Q plots of GWAS for common significant chromosomes (GGA3). The horizontal red lines indicate the thresholds for genome-wide significance (*P* < 3.7 × 10^−8^), and the horizontal blue lines indicate the thresholds for suggestive significance (*P* < 7.4 × 10^−7^)

Several common genomic regions were detected within a 0.5-Mb window, both within and across traits. In particular, chromosome GGA3 emerged as a shared region associated with immune responses to both AIV (H5 and H7) and NDV (Fig. 4). Within this region, rs316474025 and rs312870707 showed strong association signals (Additional file 3: Fig. S2a). Linkage disequilibrium (LD) analysis identified five LD blocks spanning approximately 0.89 Mb on GGA3, each comprising 2–15 SNPs (Additional file 3: Fig. S2b).

Most of the identified significant SNPs were distributed in intergenic and intronic regions of the genome. Candidate genomic regions were defined as ± 50 kb intervals flanking each significant SNP, and the genes located within these regions were annotated. Lists of candidate genes associated with AIV and NDV antibody responses are provided in Additional files 4: Tables S4 and S4, respectively.

The effects of significant SNPs were predominantly additive, explaining 13–28% of the phenotypic variance in AIV antibody responses (H5, H7, and H9) and 6–17% in NDV antibody responses (Table 3). For the AIV subtype H5, rs312870707 on GGA3 showed the strongest additive effect, accounting for 26% of the phenotypic variance. For subtype H7, rs316474025 explained 18% of the phenotypic variance, whereas rs737164663 was the top variant for subtype H9, explaining 17%. For NDV, rs318005864 on GGA27 explained 17% of the phenotypic variance and represented the most significant association in this study.

Based on their magnitude of effect, consistency across traits, and relevance to the immune response, rs316474025 (GGA3:29.39-29.49 Mb) and rs318005864 (GGA27:4.04-4.14 Mb) were prioritized for further genetic evaluation and potential application in genomic selection.

### Functional enrichment analysis

**Fig. 5:**
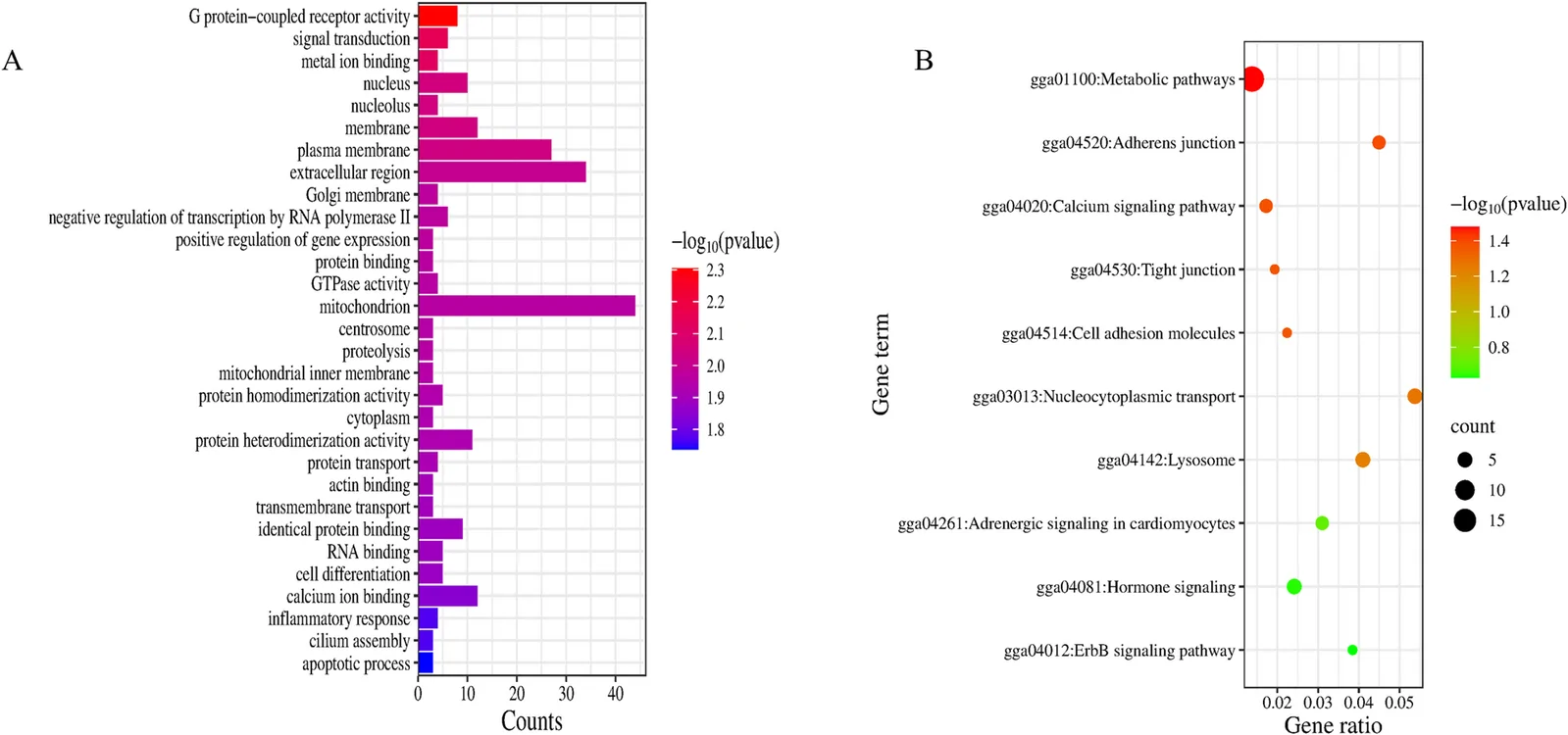
GO and KEGG enrichment analyses of 130 candidate genes. **a** GO enrichment analysis of 130 candidate genes. **a** The x-axis indicates the number of genes for each GO term, and the y-axis corresponds to the GO terms. The color of the bars represents the *P* value. **b** KEGG enrichment analysis of 130 candidate genes. The x-axis shows the gene ratio, and the y-axis represents the KEGG pathways. The dot color represents the *P* value, and the dot size represents the number of genes enriched in the reference pathway

Analyses of the identified candidate genes were significantly enriched (*P* < 0.05) (Additional file 5: Table S6). We performed Gene Ontology (GO) and KEGG enrichment analyses and identified 130 candidate genes associated with the immune response to AIV and NDV. The analyses revealed that the identified candidate genes were significantly enriched in signaling and cellular regulatory pathways rather than pathways directly involved in antibody production. The positive regulation of the MAPK cascade pathway (GO:0043410) was the significant GO term in the biological process (*P* < 0.05). The perinuclear region of the cytoplasm (GO:0048471) and protein kinase binding (GO:0019901) within cellular processes and molecular functions were the most dominant GO subcategories (Fig. 5a). In addition, six GO terms were significantly enriched: gene expression (GO:0010467), sphingolipid metabolic process (GO:0006665), cellular response to epidermal growth stimulus (GO:0071364), response to hypoxia (GO:0001666), ruffle membrane (GO:0032587), and endoplasmic reticulum membrane (GO:0005789).

Five KEGG pathways were significantly enriched: gga01100: metabolic pathways, gga04520: adherens junction, gga04020: calcium signaling pathway, gga04530: tight junction, and gga04514: cell adhesion molecules pathway (Fig. 5b), which are implicated in immune cell communication and activation. These pathways are consistent with regulatory mechanisms influencing immune responsiveness and antibody kinetics rather than direct immunoglobulin synthesis.

## Discussion

Chicken immune responses are complex traits with important economic implications [4]. Investigating the genetic basis of vaccine-induced immune responses is an important for breeding programs, as it may improve the selection of chickens with enhanced resistance or resilience to AIV and NDV [29]. Analyzing immune response traits enhances understanding of the underlying genetic architecture, thereby providing insights into poultry health and productivity. Therefore, monitoring longitudinal phenotypic changes in antibody titers is particularly important for understanding immune persistence and preventing infection. Accordingly, this study aimed to investigate the genetic basis underlying immunity persistence to AIV and NDV by integrating longitudinal antibody response profiles with time-specific GWAS in 572 F19 advanced intercross line (AIL) chickens. We detected low to moderate heritable variation and identified genomic regions associated with antibody response dynamics across the four time points (P0–P3).

Phenotypic analysis, heritability estimation, and GWAS revealed putative candidate genes, pathways, and biological functions associated with AIV and NDV immune responses. Estimated heritability for AIV ranged from 0.15 to 0.35, whereas NDV heritability was consistently moderate (0.25–0.35), higher than values reported for survival to infection (0.089) [30], but comparable to previous antibody response studies [14]. Moderate phenotypic and genetic correlations for both AIV and NDV traits indicated strong immune responsiveness, aligning with prior reports in F2 populations [14, 16]. Heritability estimates for primary NDV response in Kuchi and Tanzania Medium ecotypes (0.27 ± 0.06 and 0.29 ± 0.05) also align closely with our findings [31].

Our GWAS identified several significant SNPs that appeared as isolated signals rather than forming classical linkage disequilibrium (LD) peaks, particularly those with low minor allele frequencies (MAF) near the 0.05 threshold. Low MAF SNPs can sometimes generate spurious associations if the few carriers are overrepresented among phenotypic extremes. However, this pattern is not expected in advanced intercross line (AIL) populations, where repeated recombination events break down LD blocks, improving mapping resolution and reducing confounding from long-range LD [4]. Importantly, all analyses were conducted using a mixed linear model accounting for genomic relatedness, and only SNPs passing stringent quality control including MAF filtering, call rate, and Hardy–Weinberg equilibrium tests were retained. Therefore, while the potential limitations of low MAF SNPs are acknowledged, the observed isolated signals likely represent genuine associations.

Linear mixed model of GWAS identified 22 SNPs across 12 chromosomes, including 14 associated with AIV (H5, H7, H9) and 8 with NDV. Three SNPs rs316474025, rs737164663, and rs318005864 (on GGA3, GGA10, and GGA27) consistently influenced both AIV and NDV responses across multiple time points (P1-P3), suggesting co-regulation by multiple genetic mechanisms, consistent with prior studies in turkeys [32], who revealed different SNPs at similar time point effects of immune response during a velogenic challenge in turkeys.

Hence, antibody responses displayed antigen and time-specific genetic architectures, reflecting the complex regulation of humoral immunity. While antigen recognition is strongly influenced by the major histocompatibility complex on GGA16, antibody titers represent downstream quantitative traits modulated by multiple non-MHC loci involved in immune signaling, B-cell differentiation, cytokine regulation, and immunological memory. Temporal specificity of detected loci likely reflects phase-dependent genetic control, with early responses driven by innate and early adaptive mechanisms and later responses associated with antibody persistence. The absence of strong GGA16 signals may reflect the high polymorphism and complex structure of the MHC region, which is not fully captured by SNP-based GWAS under stringent multiple-testing correction.

Our GWAS analysis identified several SNPs with substantial effects on antibody responses. At P1, rs318005864 was significantly associated with AIV (*P* < 1.57 × 10^–7^), explaining 13% of the phenotypic variance, and at P2, it showed genome-wide significance for NDV (*P* < 2.39 × 10^–8^), accounting for 17% of the variance, indicating a strong biological impact. Similarly, rs737164663 was associated with NDV (*P* < 5.89 × 10^–8^, 11% variance) and with AIV at P3 (*P* < 6.91 × 10^–8^, 17% variance), while rs312870707 was linked to H5 antibody titers at P2, explaining 26% of the variance and 18% for H7, suggesting potential pleiotropic effects across AIV subtypes. These effect sizes are considerably higher than previously reported values (about 5%) [14, 16], highlighting the biological significance of these loci.

Several similar studies have investigated multiple genomic regions associated with the antibody response to avian infections in different chicken breeds. For example, Luo et al. [14], reported promising antibody response to AIV and NDV, and Luo, et.al. [16], identified two QTLs on GGA1 and GGA10 linked to NDV only. Yonasha et al. [33], also found two QTLs on GGA2 and GGA18, whereas Biscarini et al. [34] detected 13 QTLs mainly located on 13 chromosomes GGA3, GGA4, GGA5, GGA9, GGA13, GGA16 and GGA22. Furthermore, Luo et al. [35], revealed antibody level against infectious bronchitis virus (IBV) on five chromosomes involving GGA1, GGA3, GGA5, GGA8, and GGA9 at 1-Mb windows size in F_2_ chicken. Other recent studies reported immune response associated to fowl cholera and Eimeria in Ethiopia chicken ecotype on GGA2, GGA3 GGA20, GGA11, GGA13 and GGA27 [17]. In addition, other associations were determined on GGA1, GGA5, GGA16 and GGA24 [10]. Notably, we identified significant and unique major SNPs associated with immune responses located toward the distal regions of these chromosomes.

Furthermore, Our GWAS identified several immune-related genes located near significant SNPs, suggesting their potential involvement in modulating antibody responses to AIV and NDV in chickens. All identified candidate genes were associated with significant SNPs located within intronic regions and are directly or indirectly implicated in pathways related to humoral immune responses. For example, on GGA3 (rs316474025), we identified *NFKBIE*, *MDGA1*, and *SLC35B2* as candidate genes. *NFKBIE*, which encodes the I-κBε inhibitor of NF-κB, is critical for immune response regulation, B-cell development, and pathogen defense [36, 37]. *MDGA1* is involved in cell adhesion and migration, with known roles in neuronal development [38, 39], suggesting potential indirect effects on immune cell trafficking. *SLC35B2* encodes the PAPST1 transporter required for sulfation reactions that are essential for STING-mediated antiviral responses [40].

At GGA3 67.9–68.3 Mb (rs312870707), *ATG5* was detected, essential for autophagosome formation and MHC class II antigen presentation [41]. On GGA10 (rs737164663), nearby genes *FRMD5* and *GLCE* were associated with immune regulation and cell infiltration [42–45]. On GGA27 (rs318005864), *CDK12* and *RPL19* were identified, involved in cell cycle regulation, innate immune responses, and ribosomal function during viral infections [46, 47].

Functional annotation revealed an enrichment of broadly acting regulatory pathways rather than pathways that are directly involved in antibody production, including MAPK signaling, calcium signaling, adherens junctions, tight junctions, and metabolic pathways [8, 48, 49]. These pathways are critical for immune cell activation, intercellular communication, and the energy provisioning required for effective immune responses. The absence of enrichment for antibody production or B-cell receptor signaling pathways highlights the polygenic and regulatory nature of vaccine-induced antibody traits, which are shaped primarily by upstream signaling, cellular coordination, and immune kinetics rather than immunoglobulin synthesis alone. Similar observations have been reported in previous GWAS of immune traits, where non-canonical immune pathways contribute substantially to variation in antibody titers.

Previous studies suggest that immune activation is energetically costly and may alter nutrient partitioning and growth traits [47]. Our findings align with this concept, indicating a potential trade-off between immune function and metabolic efficiency in chickens. The concentration of immune-associated loci on GGA3, GGA10, and GGA27 further illustrates the complex and polygenic nature of immune trait regulation.

Collectively, this study provides an in-depth understanding of the genetic architecture of complex immune traits in chickens, revealing both direct and indirect regulatory mechanisms and highlighting candidate genes and pathways for further investigation in breeding programs aimed at enhancing disease resistance and productivity.

## Conclusion

In conclusion, the GWAS results significantly advanced our understanding of the genetic architecture underlying longitudinal antibody titer traits in chickens, providing valuable information for genetic selection strategies targeting immune responses to AIV and NDV, particularly during the mid and late post-vaccination periods. These traits are well defined, interpretable, and exhibit moderate heritability, supporting their potential inclusion in multi-trait selection indices.

Several significant SNPs were identified at pleiotropic loci associated with both antibody titer traits. Notably, regions on GGA3 encompassing *NFKBIE, SLC35B2*, and *ATG5*, a locus on GGA10 near *FRMD5* and *GLCE*, and a region on GGA27 containing *DDX12* and *RPL19* were consistently associated with variation in antibody titers. These genomic regions represent promising targets for genomic selection in AIL F19 chickens.

Overall, this study provides a solid genetic framework for the development of breeding strategies aimed at improving immune response traits in chickens. Future studies focusing on gene expression and validation of these loci across populations and refinement of their effects on antibody titers will further enhance their application in poultry breeding programs.

## Supplementary Information

Below is the link to the electronic supplementary material.

Supplementary Material 1

Supplementary Material 2

Supplementary Material 3

Supplementary Material 4

Supplementary Material 5

Supplementary Material 6

## Data Availability

The raw whole-genome resequencing data were deposited and are publicly available in the repository CRA043943 (https://ngdc.cncb.ac.cn/gsa/browse/CRA043943).
