# Supplementary material for: Genome-wide association study reveals putative genetic determinants of longitudinal immune responses to avian influenza and Newcastle disease in chickens
Source: Genet Sel Evol. 2026 Aug 11;58:56. doi: 10.1186/s12711-026-01077-2 (PMC13501648; doi:10.1186/s12711-026-01077-2)
Supplement: Supplementary file 1 [file 12711_2026_1077_MOESM2_ESM.pdf]

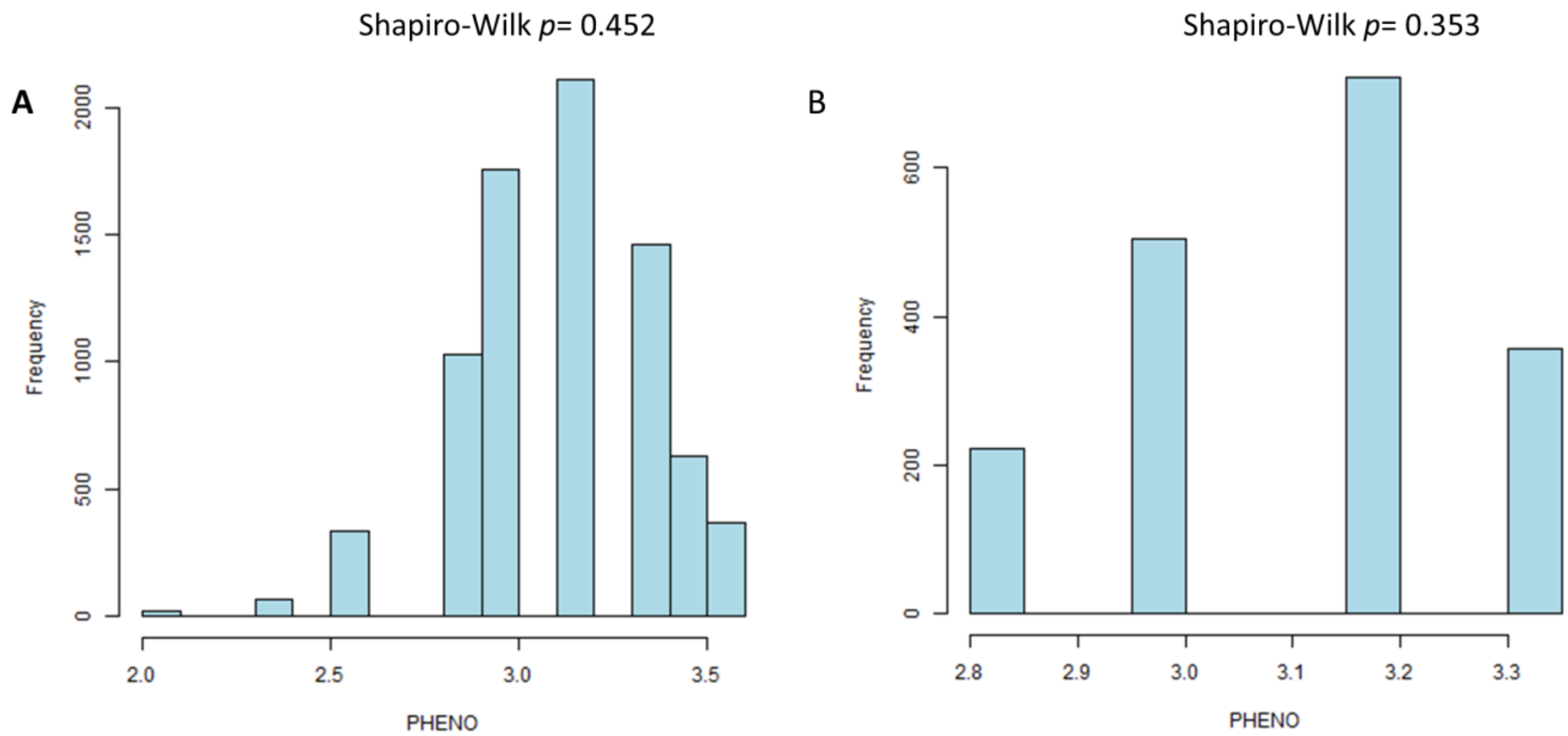
Shapiro-Wilk p= 0.452
A
Frequency
0
500
1000
1500
2000
2.0
2.5
3.0
3.5
PHENO
Shapiro-Wilk p= 0.353
B
Frequency
0
200
400
600
2.8
2.9
3.0
3.1
3.2
3.3
PHENO
