# Supplementary material for: Genome-wide association study reveals putative genetic determinants of longitudinal immune responses to avian influenza and Newcastle disease in chickens
Source: Genet Sel Evol. 2026 Aug 11;58:56. doi: 10.1186/s12711-026-01077-2 (PMC13501648; doi:10.1186/s12711-026-01077-2)
Supplement: Supplementary file 2 [file 12711_2026_1077_MOESM3_ESM.pdf]

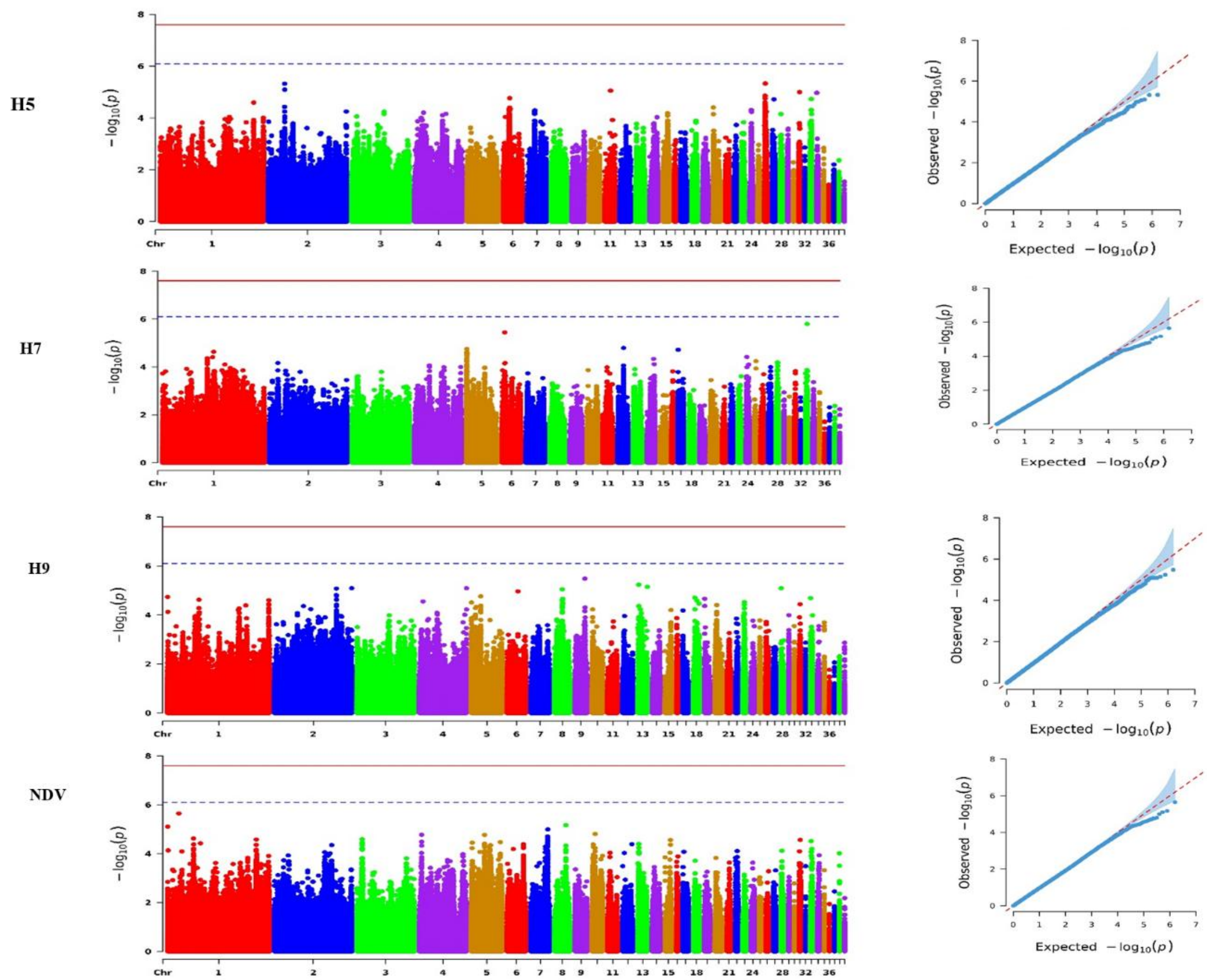
H5
H7
H9
NDV
$-\log_{10}(p)$
Chr
Observed $-\log_{10}(p)$
Expected $-\log_{10}(p)$
