# Supplementary material for: Genome-wide association study reveals putative genetic determinants of longitudinal immune responses to avian influenza and Newcastle disease in chickens
Source: Genet Sel Evol. 2026 Aug 11;58:56. doi: 10.1186/s12711-026-01077-2 (PMC13501648; doi:10.1186/s12711-026-01077-2)
Supplement: Supplementary file 3 [file 12711_2026_1077_MOESM4_ESM.pdf]

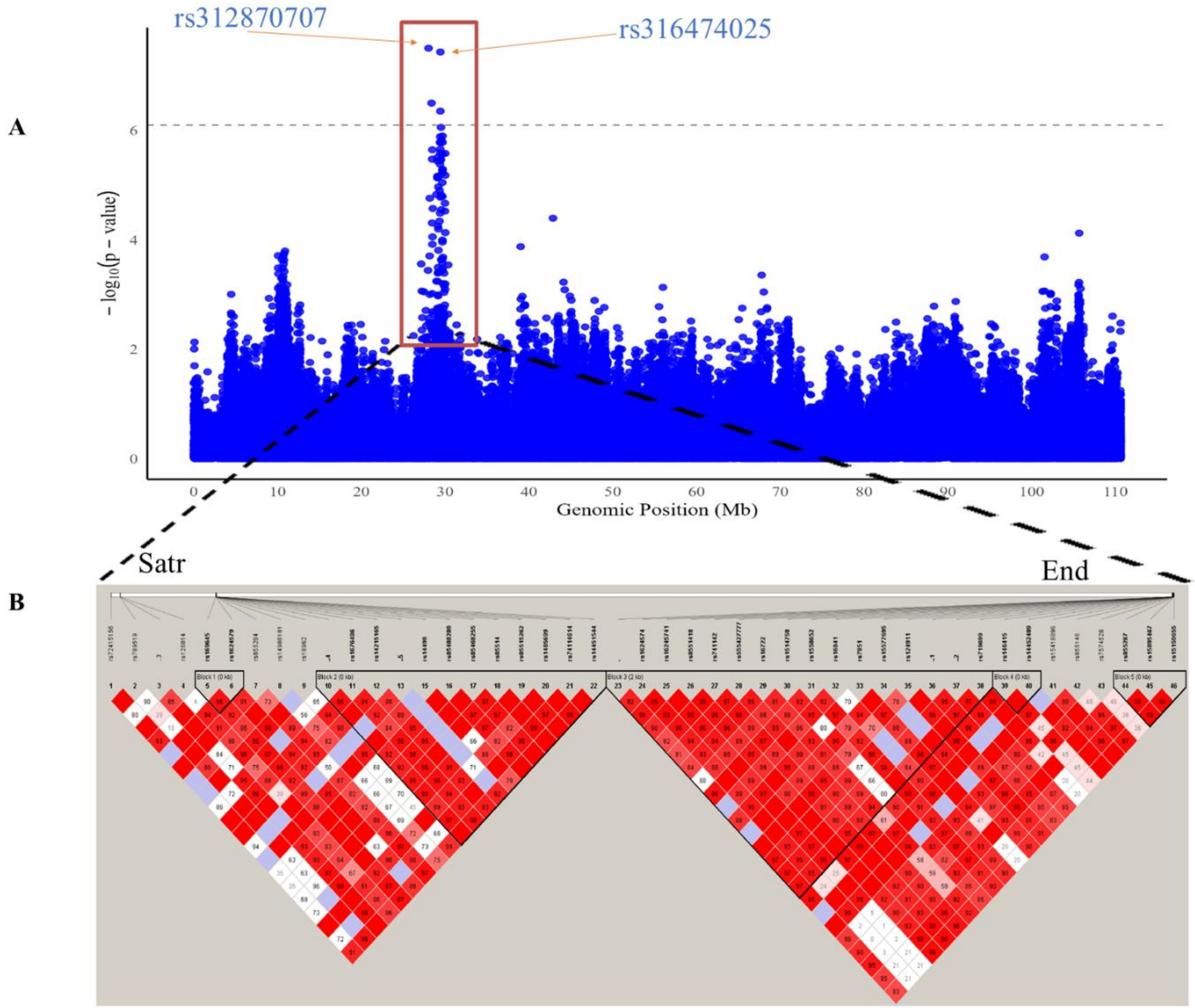
A
rs312870707
rs316474025
$-\log_{10}(p - value)$
0
2
4
6
0
10
20
30
40
50
60
70
80
90
100
110
Genomic Position (Mb)
B
Satr
End
