# Supplementary material for: Genome-wide association study reveals putative genetic determinants of longitudinal immune responses to avian influenza and Newcastle disease in chickens
Source: Genet Sel Evol. 2026 Aug 11;58:56. doi: 10.1186/s12711-026-01077-2 (PMC13501648; doi:10.1186/s12711-026-01077-2)
Supplement: Supplementary file 4 [file 12711_2026_1077_MOESM1_ESM.docx]

**Additional File 2: Table S1,**  **S2 and S3**

**Table S1. Phenotypic and genetic correlations among all AIV subtype traits studied in 572 chickens.** Phenotypic (above diagonal) and genetic correlation (below diagonal) estimates (standard errors in parentheses) across all time points

| Subtype | Comparison |  | P0 | P1 | P2 | P3 |
| --- | --- | --- | --- | --- | --- | --- |
| AIV | H5 | P0 | – | 0.07  (0.06) | 0.14  (0.07) | 0.36  (0.06) |
|  |  | P1 | 0.21  (0.5) | – | 0.38  (0.08) | 0.35  **(0.05**) |
|  |  | P2 | 0.10  (0.6) | 0.25  (0.2) | – | 0.45  (0.07) |
|  |  | P3 | 0.40  (0.6) | 0.35  (0.8) | 0.50  (0.7) | – |
|  | H7 | P0 | – | 0.25  **(0.05**) | -0.31  (**0.04**) | 0.20  (0.07) |
|  |  | P1 | 0.25  (0.08) | – | 0.25  (0.07) | 0.30  (0.07) |
|  |  | P2 | 0.20  (0.07) | 0.015  (0.07) | – | 0.35  (0.08) |
|  |  | P3 | 0.05  (0.07) | 0.025  (0.08) | 0.15  (0.06) | – |
|  | H9 | P0 | – | -0.20  (0.07) | -0.15  (**0.05**) | 0.23  (**0.05**) |
|  |  | P1 | 0.30  (0.09) | – | 0.10  (0.08) | 0.21  (0.06) |
|  |  | P2 | 0.25  (0.23) | 0.085  (0.18) | – | 0.25  (0.09) |
|  |  | P3 | 0.15  (0.4) | 0.14  (0.5) | 0.31  (0.4) | – |

**Table S2. Phenotypic and genetic correlations in antibody response to NDV traits studied in 572 chickens.** Phenotypic (above diagonal) and genetic correlation (below diagonal) estimates (standard errors in parentheses) across all time points

| **Subtype** | **Comparison** | **P0** | **P1** | **P2** | **P3** |
| --- | --- | --- | --- | --- | --- |
| NDV | P0 | – | 0.03  (**0.05**) | 0.02  (0.08) | 0.40  (**0.04**) |
|  | P1 | 0.15  (0.6) | – | 0.38  (**0.05**) | 0.54  (0.08) |
|  | P2 | 0.05  (0.52) | 0.35  (0.51) | – | 0.63  (0.07) |
|  | P3 | 0.01  (0.5) | 0.45  (0.4) | 0.52  (0.2) | – |

**Table S3.** Summary of clean reads and clean bases obtained from sequencing 587 individual samples.

| Sample ID | Clean Reads | Clean Base | Read Length | Q20(%) | Q30(%) | GC(%) |
| --- | --- | --- | --- | --- | --- | --- |
| 01211 | 40174561 | 12052368300 | PE150 | 97.9 | 93.33 | 41.41 |
| 02166 | 40115488 | 12034646400 | PE150 | 97.59 | 92.45 | 41.42 |
| 22046 | 40068602 | 12020580600 | PE150 | 97.45 | 92.01 | 41.22 |
| 40078 | 40221229 | 12066368700 | PE150 | 97.7 | 92.75 | 41.4 |
| 47111 | 40133793 | 12040137900 | PE150 | 97.09 | 90.94 | 41.35 |
| 58034 | 40098206 | 12029461800 | PE150 | 97.62 | 92.53 | 41.51 |
| 74013 | 40113244 | 12033973200 | PE150 | 97.14 | 91.09 | 41.49 |
| 80112 | 40191989 | 12057596700 | PE150 | 97.51 | 92.19 | 41.23 |
| 92292 | 40248037 | 12074411100 | PE150 | 98.08 | 93.88 | 41.57 |
| A03269 | 40151749 | 12045524700 | PE150 | 97.5 | 92.29 | 41.61 |
| A04336 | 40120166 | 12036049800 | PE150 | 97.79 | 93 | 41.65 |
| A05176 | 35412812 | 10623843600 | PE150 | 97.91 | 92.18 | 41.55 |
| A05177 | 40135103 | 12040530900 | PE150 | 97.88 | 93.32 | 41.72 |
| A10363 | 40095971 | 12028791300 | PE150 | 97.07 | 91.09 | 41.65 |
| A86331 | 40133816 | 12040144800 | PE150 | 97.61 | 92.64 | 41.9 |
| A87334 | 40091324 | 12027397200 | PE150 | 97.4 | 92.27 | 42.11 |
| A87380 | 40141377 | 12042413100 | PE150 | 97.68 | 92.68 | 41.43 |
| A87382 | 40157219 | 12047165700 | PE150 | 97.09 | 91.54 | 41.72 |
| A88153 | 40111111 | 12033333300 | PE150 | 97.29 | 91.92 | 41.66 |
| A88159 | 40096296 | 12028888800 | PE150 | 97.19 | 91.77 | 41.94 |
| A89146 | 40149636 | 12044890800 | PE150 | 97.49 | 92.27 | 41.63 |
| A90258 | 40090203 | 12027060900 | PE150 | 96.86 | 90.92 | 41.53 |
| A90263 | 40294911 | 12088473300 | PE150 | 96.96 | 91.03 | 41.87 |
| A90265 | 40099515 | 12029854500 | PE150 | 97.97 | 93.56 | 41.96 |
| A91124 | 40083447 | 12025034100 | PE150 | 97.76 | 92.92 | 41.35 |
| A93175 | 37160663 | 11148198900 | PE150 | 97.63 | 92.55 | 41.54 |
| A93252 | 40063497 | 12019049100 | PE150 | 98.68 | 95.79 | 42.38 |
| A94251 | 40024788 | 12007436400 | PE150 | 96.8 | 88.72 | 41.48 |
| B81007 | 40194679 | 12058403700 | PE150 | 98.03 | 93.79 | 41.85 |
| B81009 | 40172824 | 12051847200 | PE150 | 97.36 | 91.75 | 41.93 |
| B81013 | 40172372 | 12051711600 | PE150 | 97.85 | 91.98 | 41.37 |
| B83057 | 40269440 | 12080832000 | PE150 | 97.74 | 92.9 | 41.63 |
| B83059 | 40132859 | 12039857700 | PE150 | 96.87 | 90.94 | 41.62 |
| B84063 | 40075065 | 12022519500 | PE150 | 96.71 | 90.53 | 41.67 |
| B84064 | 40156194 | 12046858200 | PE150 | 98.27 | 94.76 | 42.71 |
| B84072 | 40100350 | 12030105000 | PE150 | 97.66 | 92.64 | 41.59 |
| B85062 | 40130308 | 12039092400 | PE150 | 97.46 | 92.2 | 41.77 |
| B92293 | 40149613 | 12044883900 | PE150 | 97.75 | 92.9 | 41.5 |
| C02169 | 39498803 | 11849640900 | PE150 | 97.41 | 91.89 | 41.29 |
| C02211 | 40013555 | 12004066500 | PE150 | 97.86 | 93.37 | 42.17 |
| D05181 | 40153346 | 12046003800 | PE150 | 97.18 | 91.21 | 41.22 |
| D07030 | 40018174 | 12005452200 | PE150 | 97.13 | 91.5 | 41.91 |
| D07034 | 40047133 | 12014139900 | PE150 | 97.56 | 92.51 | 41.71 |
| D08105 | 40209253 | 12062775900 | PE150 | 97.95 | 93.53 | 41.76 |
| D09258 | 40203421 | 12061026300 | PE150 | 97.44 | 91.96 | 41.9 |
| D11107 | 40284996 | 12085498800 | PE150 | 97.34 | 91.89 | 41.78 |
| D12059 | 40162978 | 12048893400 | PE150 | 97.24 | 91.38 | 41.35 |
| D14109 | 40057350 | 12017205000 | PE150 | 97.46 | 92 | 41.3 |
| D15081 | 40037044 | 12011113200 | PE150 | 97.95 | 93.53 | 41.77 |
| D15082 | 40083273 | 12024981900 | PE150 | 97.65 | 92.76 | 41.87 |
| D15085 | 40190480 | 12057144000 | PE150 | 96.47 | 89.94 | 41.69 |
| D16127 | 40095575 | 12028672500 | PE150 | 97.54 | 92.4 | 41.64 |
| D17056 | 40176241 | 12052872300 | PE150 | 96.84 | 88.84 | 41.38 |
| D18133 | 40156997 | 12047099100 | PE150 | 97.11 | 91.03 | 41.5 |
| D18135 | 40206447 | 12061934100 | PE150 | 97.52 | 92.38 | 41.64 |
| D18140 | 40133749 | 12040124700 | PE150 | 97.3 | 91.76 | 41.8 |
| D19036 | 40125788 | 12037736400 | PE150 | 97.21 | 91.27 | 41.36 |
| D20056 | 40228950 | 12068685000 | PE150 | 98.06 | 93.94 | 42.07 |
| D21086 | 40132663 | 12039798900 | PE150 | 97.4 | 90.84 | 41.77 |
| D21088 | 40037731 | 12011319300 | PE150 | 98.24 | 94.42 | 42.04 |
| D22039 | 40157866 | 12047359800 | PE150 | 96.59 | 90.05 | 41.49 |
| D22040 | 40146011 | 12043803300 | PE150 | 97.48 | 92.29 | 41.71 |
| D23106 | 40165220 | 12049566000 | PE150 | 97.16 | 91.53 | 41.61 |
| D23107 | 40184598 | 12055379400 | PE150 | 96.91 | 90.85 | 41.72 |
| D24112 | 40127520 | 12038256000 | PE150 | 97.44 | 91.96 | 41.25 |
| D24114 | 40133906 | 12040171800 | PE150 | 97.46 | 92.19 | 41.6 |
| D25029 | 40139598 | 12041879400 | PE150 | 98.09 | 93.95 | 41.82 |
| D25032 | 40119197 | 12035759100 | PE150 | 97.59 | 92.76 | 41.92 |
| D25035 | 40207155 | 12062146500 | PE150 | 97.12 | 91.44 | 41.67 |
| D26063 | 40236653 | 12070995900 | PE150 | 98.43 | 95 | 42.67 |
| D27136 | 38400692 | 11520207600 | PE150 | 97.59 | 92.35 | 41.51 |
| D28110 | 40136970 | 12041091000 | PE150 | 97.76 | 92.92 | 41.56 |
| D29105 | 40157313 | 12047193900 | PE150 | 97.36 | 91.92 | 41.65 |
| D30114 | 40106725 | 12032017500 | PE150 | 97.8 | 93.08 | 41.6 |
| D31061 | 38594805 | 11578441500 | PE150 | 97.36 | 91.76 | 41.45 |
| D31063 | 40159989 | 12047996700 | PE150 | 98.19 | 93.23 | 41.51 |
| D32068 | 40178505 | 12053551500 | PE150 | 97.03 | 90.79 | 41.34 |
| D33108 | 40169146 | 12050743800 | PE150 | 96.82 | 90.81 | 41.61 |
| D33109 | 40240457 | 12072137100 | PE150 | 97.19 | 91.65 | 41.72 |
| D34114 | 40168237 | 12050471100 | PE150 | 97.64 | 92.68 | 41.57 |
| D34117 | 40037705 | 12011311500 | PE150 | 98.06 | 93.87 | 41.71 |
| D34122 | 40138731 | 12041619300 | PE150 | 98.59 | 94.8 | 41.9 |
| D34124 | 40095628 | 12028688400 | PE150 | 96.95 | 91.16 | 41.58 |
| D35117 | 40085073 | 12025521900 | PE150 | 97.42 | 92.06 | 41.46 |
| D37062 | 40181317 | 12054395100 | PE150 | 97.12 | 91.45 | 41.5 |
| D37063 | 40221172 | 12066351600 | PE150 | 97.66 | 92.63 | 41.43 |
| D37071 | 40245093 | 12073527900 | PE150 | 97.95 | 93.51 | 41.62 |
| D38059 | 40081836 | 12024550800 | PE150 | 97.4 | 91.85 | 41.53 |
| D39056 | 40049915 | 12014974500 | PE150 | 98.14 | 93.02 | 41.64 |
| D39057 | 40143812 | 12043143600 | PE150 | 97.15 | 91.72 | 41.82 |
| D39058 | 40189938 | 12056981400 | PE150 | 96.97 | 91.23 | 41.82 |
| D40085 | 40022222 | 12006666600 | PE150 | 97.75 | 92.91 | 41.39 |
| D41057 | 40280645 | 12084193500 | PE150 | 97.5 | 92.39 | 41.93 |
| D42136 | 40187339 | 12056201700 | PE150 | 97.33 | 91.84 | 41.82 |
| D43129 | 40172312 | 12051693600 | PE150 | 97.43 | 92.11 | 41.54 |
| D43133 | 40232056 | 12069616800 | PE150 | 96.42 | 89.77 | 41.68 |
| D44110 | 40158782 | 12047634600 | PE150 | 97.36 | 91.72 | 41.49 |
| D44112 | 40103238 | 12030971400 | PE150 | 97.55 | 92.31 | 41.49 |
| D44114 | 40166232 | 12049869600 | PE150 | 98.02 | 93.75 | 41.61 |
| D45032 | 40121279 | 12036383700 | PE150 | 98.42 | 94.84 | 42.46 |
| D46141 | 40214984 | 12064495200 | PE150 | 98.11 | 93.98 | 41.78 |
| D46143 | 39211800 | 11763540000 | PE150 | 96.97 | 90.86 | 41.54 |
| D46145 | 40128104 | 12038431200 | PE150 | 97.22 | 91.72 | 41.79 |
| D47114 | 40181951 | 12054585300 | PE150 | 98.1 | 93.98 | 41.71 |
| D47118 | 39640875 | 11892262500 | PE150 | 96.94 | 91 | 41.69 |
| D47119 | 40206750 | 12062025000 | PE150 | 97.3 | 91.53 | 41.85 |
| D47122 | 40213084 | 12063925200 | PE150 | 97.06 | 91.49 | 41.74 |
| D48138 | 40220458 | 12066137400 | PE150 | 97.79 | 93.03 | 41.49 |
| D48140 | 40156721 | 12047016300 | PE150 | 97.28 | 91.74 | 41.69 |
| D49134 | 40048855 | 12014656500 | PE150 | 97.42 | 92.1 | 41.78 |
| D49135 | 36742774 | 11022832200 | PE150 | 96.64 | 88 | 41.37 |
| D51131 | 40198358 | 12059507400 | PE150 | 97.62 | 92.51 | 41.43 |
| D51138 | 37940794 | 11382238200 | PE150 | 96.84 | 90.87 | 41.95 |
| D52029 | 40237311 | 12071193300 | PE150 | 97.87 | 93.4 | 41.86 |
| D52030 | 40199880 | 12059964000 | PE150 | 97.82 | 93.14 | 41.77 |
| D52033 | 40126872 | 12038061600 | PE150 | 97 | 90.72 | 41.25 |
| D53056 | 40114131 | 12034239300 | PE150 | 98.57 | 95.42 | 42.53 |
| D54110 | 40278113 | 12083433900 | PE150 | 97.59 | 92.55 | 41.61 |
| D54111 | 40168164 | 12050449200 | PE150 | 98.12 | 94.04 | 41.97 |
| D54113 | 40178619 | 12053585700 | PE150 | 98.2 | 94.33 | 41.89 |
| D55054 | 40133694 | 12040108200 | PE150 | 97.59 | 92.38 | 42.02 |
| D56056 | 40108205 | 12032461500 | PE150 | 97.61 | 92.61 | 41.77 |
| D57109 | 40044106 | 12013231800 | PE150 | 96.73 | 90.38 | 41.55 |
| D57116 | 40049771 | 12014931300 | PE150 | 96.83 | 90.86 | 41.78 |
| D59104 | 40213984 | 12064195200 | PE150 | 98.01 | 93.68 | 41.59 |
| D59105 | 40152833 | 12045849900 | PE150 | 96.7 | 90.51 | 41.61 |
| D59110 | 40046206 | 12013861800 | PE150 | 97.34 | 91.87 | 41.62 |
| D60103 | 40111124 | 12033337200 | PE150 | 96.81 | 90.62 | 41.67 |
| D60106 | 34998438 | 10499531400 | PE150 | 96.75 | 88.44 | 41.57 |
| D62133 | 40124034 | 12037210200 | PE150 | 97.33 | 91.76 | 41.42 |
| D64109 | 40054901 | 12016470300 | PE150 | 98.03 | 92.64 | 41.59 |
| D64111 | 40265239 | 12079571700 | PE150 | 97.56 | 92.29 | 41.26 |
| D65020 | 40060311 | 12018093300 | PE150 | 96.9 | 90.39 | 41.39 |
| D65021 | 40226862 | 12068058600 | PE150 | 97.66 | 92.8 | 41.95 |
| D66055 | 40025478 | 12007643400 | PE150 | 97.11 | 91.43 | 41.62 |
| D66058 | 40119012 | 12035703600 | PE150 | 97.6 | 92.48 | 41.46 |
| D67137 | 38384860 | 11515458000 | PE150 | 96.54 | 87.69 | 41.35 |
| D68140 | 40071952 | 12021585600 | PE150 | 97.55 | 92.27 | 42.08 |
| D69018 | 40283808 | 12085142400 | PE150 | 97.64 | 92.56 | 41.53 |
| D70113 | 40143139 | 12042941700 | PE150 | 97.61 | 92.5 | 41.5 |
| D71059 | 40200263 | 12060078900 | PE150 | 96.72 | 90.54 | 41.67 |
| D71061 | 40061291 | 12018387300 | PE150 | 97.94 | 93.62 | 42.07 |
| D71062 | 40296984 | 12089095200 | PE150 | 97.76 | 93.04 | 41.86 |
| D72016 | 40151746 | 12045523800 | PE150 | 98.05 | 93.84 | 41.88 |
| D72128 | 40239790 | 12071937000 | PE150 | 98 | 93.7 | 41.92 |
| D72129 | 40167702 | 12050310600 | PE150 | 97.73 | 92.87 | 41.75 |
| D72134 | 40118229 | 12035468700 | PE150 | 97.53 | 92.42 | 41.83 |
| D72139 | 40216402 | 12064920600 | PE150 | 97.55 | 92.31 | 41.37 |
| D73017 | 40170621 | 12051186300 | PE150 | 97.25 | 91.63 | 41.69 |
| D73019 | 40221274 | 12066382200 | PE150 | 98.02 | 93.77 | 41.95 |
| D74017 | 40236360 | 12070908000 | PE150 | 96.69 | 90.48 | 41.75 |
| D74019 | 40207855 | 12062356500 | PE150 | 96.63 | 90.3 | 41.71 |
| D74024 | 40064169 | 12019250700 | PE150 | 96.52 | 90.06 | 41.54 |
| D74102 | 40199760 | 12059928000 | PE150 | 96.88 | 90.75 | 41.4 |
| D75103 | 40138655 | 12041596500 | PE150 | 96.97 | 91.08 | 41.76 |
| D75106 | 40040802 | 12012240600 | PE150 | 97.61 | 92.5 | 41.5 |
| D76031 | 40301560 | 12090468000 | PE150 | 97.28 | 91.72 | 41.76 |
| D76054 | 38094051 | 11428215300 | PE150 | 97.5 | 92.19 | 41.62 |
| D76055 | 40075441 | 12022632300 | PE150 | 97.47 | 92.21 | 41.68 |
| D76061 | 40139513 | 12041853900 | PE150 | 98.12 | 92.91 | 41.58 |
| D76063 | 40084672 | 12025401600 | PE150 | 97.3 | 91.54 | 41.45 |
| D76067 | 40263919 | 12079175700 | PE150 | 97.43 | 92.17 | 41.88 |
| D77033 | 39969004 | 11990701200 | PE150 | 97.18 | 91.13 | 41.99 |
| D79106 | 40243396 | 12073018800 | PE150 | 97.62 | 92.5 | 41.19 |
| D85064 | 40105723 | 12031716900 | PE150 | 97.48 | 92.11 | 41.28 |
| D39065 | 40082515 | 12024754500 | PE150 | 97.16 | 93.74 | 42.27 |
| D51133 | 40107915 | 12032374500 | PE150 | 96.96 | 93.33 | 42.59 |
| A01220 | 40112401 | 12033720300 | PE150 | 96.36 | 89.85 | 42.24 |
| A03266 | 40179579 | 12053873700 | PE150 | 96.5 | 90.28 | 42.3 |
| A03267 | 40057059 | 12017117700 | PE150 | 96.46 | 90.05 | 42.42 |
| A03268 | 40127530 | 12038259000 | PE150 | 98.72 | 95.62 | 42.37 |
| A03270 | 40146679 | 12044003700 | PE150 | 97.9 | 93.22 | 41.89 |
| A03342 | 40137459 | 12041237700 | PE150 | 98.83 | 95.99 | 42.44 |
| A03345 | 40133316 | 12039994800 | PE150 | 96.52 | 89.91 | 42.04 |
| A04332 | 39257899 | 11777369700 | PE150 | 95.9 | 88.64 | 41.95 |
| A04335 | 40160347 | 12048104100 | PE150 | 95.78 | 88.78 | 42.06 |
| A04338 | 40115835 | 12034750500 | PE150 | 97.44 | 92 | 42.22 |
| A04340 | 37046909 | 11114072700 | PE150 | 96.81 | 90.6 | 42.25 |
| A04341 | 40035854 | 12010756200 | PE150 | 96.45 | 89.82 | 41.77 |
| A04343 | 39747799 | 11924339700 | PE150 | 96.47 | 90.13 | 42.11 |
| A05172 | 37103064 | 11130919200 | PE150 | 96.1 | 89.35 | 42.45 |
| A05174 | 36083421 | 10825026300 | PE150 | 96.67 | 90.21 | 42.36 |
| A05175 | 40104364 | 12031309200 | PE150 | 96.56 | 90.06 | 41.64 |
| A05178 | 40127224 | 12038167200 | PE150 | 97.51 | 92.19 | 42.18 |
| A05179 | 40195114 | 12058534200 | PE150 | 96.44 | 89.69 | 41.99 |
| A05183 | 40073465 | 12022039500 | PE150 | 97.57 | 92.37 | 42.23 |
| A09257 | 40210036 | 12063010800 | PE150 | 96.78 | 90.47 | 41.67 |
| A09261 | 40150442 | 12045132600 | PE150 | 98.23 | 94.36 | 41.66 |
| A09265 | 40068710 | 12020613000 | PE150 | 97.89 | 93.18 | 41.95 |
| A09266 | 40165928 | 12049778400 | PE150 | 97.86 | 93.26 | 41.93 |
| A09267 | 36736497 | 11020949100 | PE150 | 95.47 | 88.17 | 41.92 |
| A09272 | 40255643 | 12076692900 | PE150 | 96.39 | 89.95 | 42.07 |
| A09274 | 40114310 | 12034293000 | PE150 | 98.17 | 94.09 | 42 |
| A10326 | 40198584 | 12059575200 | PE150 | 97.81 | 93.1 | 41.83 |
| A10327 | 40105289 | 12031586700 | PE150 | 96.96 | 90.98 | 41.87 |
| A10330 | 40152517 | 12045755100 | PE150 | 96.87 | 90.75 | 42.19 |
| A10353 | 39764210 | 11929263000 | PE150 | 96.93 | 90.95 | 42.31 |
| A10355 | 40196991 | 12059097300 | PE150 | 98.46 | 94.88 | 42.14 |
| A10358 | 40152832 | 12045849600 | PE150 | 96.1 | 89.2 | 42.38 |
| A10359 | 40176790 | 12053037000 | PE150 | 97.83 | 92.96 | 41.95 |
| A10361 | 40219821 | 12065946300 | PE150 | 98.15 | 94.01 | 41.72 |
| A10362 | 40126920 | 12038076000 | PE150 | 96.52 | 89.74 | 42.06 |
| A10382 | 40235471 | 12070641300 | PE150 | 96.78 | 90.47 | 41.7 |
| A69263 | 40171228 | 12051368400 | PE150 | 98.45 | 94.85 | 42.46 |
| A85053 | 40214972 | 12064491600 | PE150 | 96.94 | 90.96 | 41.98 |
| A86173 | 40119698 | 12035909400 | PE150 | 97.31 | 91.57 | 42.16 |
| A86174 | 40162990 | 12048897000 | PE150 | 96.78 | 90.51 | 42.21 |
| A86175 | 40102999 | 12030899700 | PE150 | 96.85 | 90.67 | 42.25 |
| A86326 | 40241009 | 12072302700 | PE150 | 98 | 93.66 | 42.08 |
| A86328 | 40168100 | 12050430000 | PE150 | 98.06 | 93.74 | 42 |
| A86330 | 40183344 | 12055003200 | PE150 | 97.88 | 93.28 | 41.95 |
| A86332 | 40250249 | 12075074700 | PE150 | 96.23 | 89.22 | 41.85 |
| A86334 | 40110251 | 12033075300 | PE150 | 96.69 | 90.13 | 41.98 |
| A86335 | 40208763 | 12062628900 | PE150 | 98.77 | 95.83 | 42.61 |
| A86336 | 40209833 | 12062949900 | PE150 | 96.53 | 89.78 | 42.02 |
| A86338 | 40164051 | 12049215300 | PE150 | 98.72 | 95.55 | 42.02 |
| A86339 | 38449254 | 11534776200 | PE150 | 95.6 | 87.87 | 41.92 |
| A86340 | 40238413 | 12071523900 | PE150 | 96.34 | 89.84 | 42.26 |
| A87331 | 40148549 | 12044564700 | PE150 | 96.11 | 89.23 | 42.51 |
| A87332 | 40260550 | 12078165000 | PE150 | 96.6 | 90.53 | 42.3 |
| A87333 | 40179481 | 12053844300 | PE150 | 97.43 | 91.98 | 41.99 |
| A87335 | 40171567 | 12051470100 | PE150 | 96.39 | 89.95 | 42.23 |
| A87336 | 40265830 | 12079749000 | PE150 | 97.6 | 92.4 | 41.83 |
| A87337 | 40153152 | 12045945600 | PE150 | 97.44 | 91.99 | 42.19 |
| A87338 | 40113089 | 12033926700 | PE150 | 95.78 | 88.59 | 41.99 |
| A87339 | 40194970 | 12058491000 | PE150 | 96.61 | 90.23 | 41.85 |
| A87340 | 40095597 | 12028679100 | PE150 | 95.88 | 88.77 | 42 |
| A87376 | 40057772 | 12017331600 | PE150 | 96.25 | 89.16 | 41.69 |
| A87379 | 40037026 | 12011107800 | PE150 | 98.12 | 93.87 | 41.4 |
| A87384 | 40257649 | 12077294700 | PE150 | 97.87 | 93.29 | 42.1 |
| A88151 | 40193411 | 12058023300 | PE150 | 96.4 | 89.57 | 41.97 |
| A88152 | 40180549 | 12054164700 | PE150 | 95.89 | 88.62 | 41.95 |
| A88157 | 40139602 | 12041880600 | PE150 | 97.49 | 92.24 | 41.9 |
| A89145 | 40119294 | 12035788200 | PE150 | 96.73 | 90.44 | 42.28 |
| A90255 | 40089420 | 12026826000 | PE150 | 97.56 | 92.37 | 41.95 |
| A90256 | 40145281 | 12043584300 | PE150 | 96.56 | 89.83 | 41.75 |
| A90259 | 40193158 | 12057947400 | PE150 | 96.71 | 90.26 | 41.65 |
| A90260 | 40271322 | 12081396600 | PE150 | 96.81 | 90.78 | 42.12 |
| A90262 | 38779485 | 11633845500 | PE150 | 96.84 | 90.69 | 42.26 |
| A90264 | 40136835 | 12041050500 | PE150 | 98.02 | 93.56 | 41.67 |
| A91122 | 36903052 | 11070915600 | PE150 | 96.2 | 89.43 | 42.37 |
| A91123 | 40123045 | 12036913500 | PE150 | 96.27 | 89.41 | 41.78 |
| A91279 | 40018637 | 12005591100 | PE150 | 96.67 | 90.22 | 41.88 |
| A93251 | 40134876 | 12040462800 | PE150 | 98.1 | 93.84 | 41.83 |
| B81008 | 40234613 | 12070383900 | PE150 | 96.17 | 89.37 | 41.85 |
| B81011 | 40119238 | 12035771400 | PE150 | 97.47 | 92.09 | 42.02 |
| B81012 | 40112314 | 12033694200 | PE150 | 96.96 | 90.88 | 41.94 |
| B82003 | 40075370 | 12022611000 | PE150 | 96.55 | 89.99 | 42.1 |
| B82007 | 40162552 | 12048765600 | PE150 | 98.62 | 95.43 | 42.4 |
| B82008 | 40104259 | 12031277700 | PE150 | 97.77 | 92.94 | 41.71 |
| B82010 | 40267555 | 12080266500 | PE150 | 96.63 | 90.01 | 41.62 |
| B83054 | 40065375 | 12019612500 | PE150 | 96.88 | 90.81 | 41.99 |
| B83055 | 40028336 | 12008500800 | PE150 | 96.76 | 90.4 | 41.7 |
| B83058 | 40113528 | 12034058400 | PE150 | 96.38 | 90.04 | 42.23 |
| B83060 | 40201954 | 12060586200 | PE150 | 97.34 | 92.09 | 42.55 |
| B83061 | 40175359 | 12052607700 | PE150 | 98.54 | 95.29 | 42.66 |
| B84057 | 40250040 | 12075012000 | PE150 | 95.69 | 88.11 | 42.14 |
| B84059 | 40155424 | 12046627200 | PE150 | 97.52 | 92.67 | 42.64 |
| B84061 | 40218176 | 12065452800 | PE150 | 95.49 | 87.54 | 42.02 |
| B84062 | 40111532 | 12033459600 | PE150 | 98.05 | 93.7 | 41.72 |
| B84066 | 40079796 | 12023938800 | PE150 | 96.6 | 90.61 | 42.72 |
| B84068 | 40255004 | 12076501200 | PE150 | 96.29 | 89.78 | 42.19 |
| B84069 | 40107267 | 12032180100 | PE150 | 96.19 | 89.54 | 42.09 |
| B84070 | 40159456 | 12047836800 | PE150 | 96.34 | 90.22 | 41.86 |
| B84076 | 40143543 | 12043062900 | PE150 | 95.69 | 88.09 | 41.99 |
| B84278 | 40123097 | 12036929100 | PE150 | 97.88 | 93.22 | 42.08 |
| B85051 | 40196106 | 12058831800 | PE150 | 98.5 | 95.01 | 42.14 |
| B85054 | 40203429 | 12061028700 | PE150 | 96.62 | 90.04 | 41.87 |
| B85055 | 40166828 | 12050048400 | PE150 | 97.71 | 92.62 | 41.42 |
| B85058 | 40279907 | 12083972100 | PE150 | 96.4 | 89.79 | 42.16 |
| B85065 | 40188903 | 12056670900 | PE150 | 96.47 | 89.63 | 42.05 |
| B85066 | 40127986 | 12038395800 | PE150 | 96.63 | 90.11 | 42.15 |
| B85068 | 40115575 | 12034672500 | PE150 | 96.67 | 90.21 | 42.13 |
| B92291 | 40120790 | 12036237000 | PE150 | 96.34 | 89.89 | 42.34 |
| C01212 | 40144574 | 12043372200 | PE150 | 96.48 | 89.77 | 42.12 |
| C01218 | 39558682 | 11867604600 | PE150 | 96.35 | 89.58 | 41.74 |
| C01219 | 40230782 | 12069234600 | PE150 | 97.18 | 92.02 | 42.59 |
| C02161 | 40061893 | 12018567900 | PE150 | 98.7 | 95.52 | 42.36 |
| C02163 | 38508595 | 11552578500 | PE150 | 95.69 | 88.07 | 41.87 |
| C02164 | 40129525 | 12038857500 | PE150 | 98 | 93.49 | 41.51 |
| C02168 | 40154709 | 12046412700 | PE150 | 97.42 | 91.94 | 42.2 |
| C02172 | 40147675 | 12044302500 | PE150 | 96.48 | 90.02 | 41.71 |
| C02376 | 40222512 | 12066753600 | PE150 | 96.18 | 89.05 | 41.92 |
| C02378 | 40095669 | 12028700700 | PE150 | 95.98 | 89.04 | 41.99 |
| C64108 | 40151539 | 12045461700 | PE150 | 96.13 | 89.65 | 41.73 |
| D01213 | 40141123 | 12042336900 | PE150 | 96.43 | 89.78 | 41.96 |
| D03346 | 40057969 | 12017390700 | PE150 | 95.77 | 88.8 | 42.01 |
| D07032 | 40174058 | 12052217400 | PE150 | 97.92 | 93.35 | 41.99 |
| D07037 | 40038700 | 12011610000 | PE150 | 96.55 | 90.36 | 42.14 |
| D07039 | 40123915 | 12037174500 | PE150 | 96.64 | 89.97 | 41.94 |
| D07042 | 40196931 | 12059079300 | PE150 | 96.55 | 89.72 | 41.99 |
| D08104 | 40070099 | 12021029700 | PE150 | 95.95 | 88.97 | 42.06 |
| D08106 | 40138969 | 12041690700 | PE150 | 97.62 | 92.95 | 42.42 |
| D08109 | 40112581 | 12033774300 | PE150 | 96.2 | 89.45 | 42.37 |
| D08110 | 40116502 | 12034950600 | PE150 | 98.82 | 95.97 | 42.48 |
| D08111 | 40061928 | 12018578400 | PE150 | 98.81 | 95.89 | 42.34 |
| D08112 | 40164927 | 12049478100 | PE150 | 97.79 | 92.94 | 41.87 |
| D08113 | 37326275 | 11197882500 | PE150 | 96.98 | 91.07 | 42.28 |
| D08114 | 40155785 | 12046735500 | PE150 | 96.35 | 89.26 | 41.64 |
| D11106 | 40135837 | 12040751100 | PE150 | 96.09 | 89.57 | 42.32 |
| D11108 | 40235400 | 12070620000 | PE150 | 96.47 | 89.83 | 42.03 |
| D11109 | 40222098 | 12066629400 | PE150 | 96.6 | 90.24 | 42.11 |
| D11110 | 40146003 | 12043800900 | PE150 | 98.75 | 95.7 | 42.3 |
| D11115 | 40091408 | 12027422400 | PE150 | 96.18 | 89.41 | 42.43 |
| D11116 | 40165023 | 12049506900 | PE150 | 95.79 | 88.36 | 41.9 |
| D11117 | 40210302 | 12063090600 | PE150 | 96.87 | 90.72 | 42.28 |
| D11118 | 40244136 | 12073240800 | PE150 | 96.75 | 90.35 | 41.61 |
| D11119 | 40238781 | 12071634300 | PE150 | 97.72 | 92.79 | 41.85 |
| D11120 | 40119947 | 12035984100 | PE150 | 96.88 | 91.21 | 42.14 |
| D12051 | 40127175 | 12038152500 | PE150 | 96.32 | 89.33 | 41.97 |
| D12052 | 40073251 | 12021975300 | PE150 | 96.01 | 88.93 | 42.2 |
| D12055 | 40157150 | 12047145000 | PE150 | 98.1 | 93.86 | 41.85 |
| D12056 | 40059570 | 12017871000 | PE150 | 96.6 | 89.86 | 41.98 |
| D12057 | 33237931 | 9971379300 | PE150 | 96.93 | 90.93 | 42.34 |
| D12058 | 40125153 | 12037545900 | PE150 | 97.5 | 92.15 | 41.98 |
| D12062 | 40179479 | 12053843700 | PE150 | 96.78 | 90.58 | 42.15 |
| D12063 | 40118810 | 12035643000 | PE150 | 95.71 | 88.66 | 41.88 |
| D13077 | 40148442 | 12044532600 | PE150 | 97.58 | 92.31 | 41.75 |
| D13078 | 40265922 | 12079776600 | PE150 | 96.8 | 90.47 | 42.1 |
| D14110 | 40098441 | 12029532300 | PE150 | 97.97 | 93.48 | 41.75 |
| D14111 | 40060067 | 12018020100 | PE150 | 96.83 | 90.64 | 41.73 |
| D14113 | 40141748 | 12042524400 | PE150 | 96.41 | 90.01 | 41.98 |
| D15038 | 40075123 | 12022536900 | PE150 | 96.71 | 90.25 | 41.45 |
| D15080 | 40036184 | 12010855200 | PE150 | 96.73 | 90.83 | 42.3 |
| D16130 | 40025262 | 12007578600 | PE150 | 96.45 | 89.84 | 42.06 |
| D16131 | 40091989 | 12027596700 | PE150 | 96.36 | 89.66 | 41.96 |
| D16134 | 40070643 | 12021192900 | PE150 | 96.93 | 90.95 | 42.2 |
| D17053 | 39069732 | 11720919600 | PE150 | 96.34 | 89.6 | 41.79 |
| D17055 | 40174770 | 12052431000 | PE150 | 98.1 | 93.85 | 42.1 |
| D17552 | 40064354 | 12019306200 | PE150 | 96.14 | 89.27 | 42.23 |
| D18130 | 40113696 | 12034108800 | PE150 | 97.83 | 93.12 | 41.81 |
| D18139 | 40174703 | 12052410900 | PE150 | 98.91 | 96.32 | 42.78 |
| D18141 | 40090378 | 12027113400 | PE150 | 98.73 | 95.64 | 42.25 |
| D19013 | 40173083 | 12051924900 | PE150 | 96.36 | 89.5 | 41.62 |
| D19035 | 40219876 | 12065962800 | PE150 | 98.08 | 93.76 | 41.52 |
| D19038 | 40263694 | 12079108200 | PE150 | 96.29 | 89.45 | 41.75 |
| D19042 | 40181381 | 12054414300 | PE150 | 97.53 | 92.27 | 42.34 |
| D19043 | 40181681 | 12054504300 | PE150 | 98.72 | 95.62 | 42.3 |
| D20053 | 39656647 | 11896994100 | PE150 | 96.89 | 91.25 | 42.2 |
| D20054 | 40111816 | 12033544800 | PE150 | 96.36 | 89.59 | 41.96 |
| D20057 | 39431818 | 11829545400 | PE150 | 96.05 | 88.61 | 41.74 |
| D20058 | 40150992 | 12045297600 | PE150 | 96.62 | 90.51 | 42.39 |
| D20060 | 40121421 | 12036426300 | PE150 | 98.57 | 95.28 | 42.57 |
| D20061 | 40133077 | 12039923100 | PE150 | 96.68 | 90.23 | 42.09 |
| D21076 | 40125522 | 12037656600 | PE150 | 96.73 | 90.33 | 41.7 |
| D21077 | 40228460 | 12068538000 | PE150 | 96.24 | 89.56 | 41.84 |
| D21078 | 40116923 | 12035076900 | PE150 | 97.46 | 92.2 | 41.91 |
| D21080 | 40123083 | 12036924900 | PE150 | 96.9 | 90.82 | 41.72 |
| D21084 | 40063705 | 12019111500 | PE150 | 96.4 | 89.71 | 40.92 |
| D21090 | 40056694 | 12017008200 | PE150 | 96.65 | 90.64 | 42.25 |
| D22033 | 40178835 | 12053650500 | PE150 | 96.68 | 90.14 | 41.51 |
| D22034 | 40140550 | 12042165000 | PE150 | 96.66 | 90.09 | 42.23 |
| D22036 | 40097381 | 12029214300 | PE150 | 96.1 | 89.63 | 41.95 |
| D22037 | 40109515 | 12032854500 | PE150 | 96.35 | 89.62 | 42.12 |
| D22044 | 40174916 | 12052474800 | PE150 | 95.55 | 87.71 | 42.08 |
| D22047 | 40081923 | 12024576900 | PE150 | 96.59 | 90.81 | 41.95 |
| D23105 | 40228000 | 12068400000 | PE150 | 97.05 | 91.25 | 42.41 |
| D23108 | 36658968 | 10997690400 | PE150 | 96.55 | 90.21 | 42.49 |
| D23109 | 40255616 | 12076684800 | PE150 | 96.11 | 88.77 | 41.75 |
| D24104 | 40208025 | 12062407500 | PE150 | 96.5 | 89.91 | 41.88 |
| D24107 | 40174744 | 12052423200 | PE150 | 97.84 | 93.17 | 42.03 |
| D24108 | 40283569 | 12085070700 | PE150 | 96.7 | 90.78 | 42.13 |
| D24117 | 40258643 | 12077592900 | PE150 | 96.37 | 89.85 | 42.43 |
| D24119 | 40126761 | 12038028300 | PE150 | 97.97 | 93.54 | 41.89 |
| D24122 | 40225818 | 12067745400 | PE150 | 97.85 | 93.17 | 42.08 |
| D24123 | 40236432 | 12070929600 | PE150 | 95.83 | 88.47 | 42.08 |
| D25033 | 40170478 | 12051143400 | PE150 | 96.59 | 90.47 | 42.17 |
| D25034 | 40180255 | 12054076500 | PE150 | 95.81 | 88.4 | 42 |
| D25037 | 40248937 | 12074681100 | PE150 | 97.25 | 91.56 | 41.94 |
| D26057 | 40110822 | 12033246600 | PE150 | 98.13 | 94.03 | 41.94 |
| D26060 | 40127644 | 12038293200 | PE150 | 96.52 | 89.8 | 42.19 |
| D26061 | 40225301 | 12067590300 | PE150 | 96.41 | 89.73 | 41.35 |
| D26062 | 40284592 | 12085377600 | PE150 | 96.36 | 89.46 | 42 |
| D27133 | 40195160 | 12058548000 | PE150 | 97.21 | 91.91 | 42.8 |
| D27138 | 40257065 | 12077119500 | PE150 | 97.9 | 93.31 | 42.05 |
| D28106 | 40047009 | 12014102700 | PE150 | 96.61 | 90.03 | 41.72 |
| D28107 | 40055994 | 12016798200 | PE150 | 98.16 | 94.05 | 41.97 |
| D28111 | 40223500 | 12067050000 | PE150 | 98.03 | 93.66 | 41.85 |
| D28113 | 40122359 | 12036707700 | PE150 | 96.64 | 90 | 42.16 |
| D28114 | 40083194 | 12024958200 | PE150 | 95.9 | 89.1 | 42 |
| D30112 | 40134575 | 12040372500 | PE150 | 98.66 | 95.35 | 41.99 |
| D30113 | 40114401 | 12034320300 | PE150 | 96 | 88.93 | 42.32 |
| D31060 | 40113546 | 12034063800 | PE150 | 97.08 | 91.28 | 42.32 |
| D31064 | 40162639 | 12048791700 | PE150 | 96.57 | 90.11 | 41.86 |
| D31068 | 40289077 | 12086723100 | PE150 | 97.25 | 91.56 | 41.89 |
| D31070 | 40272510 | 12081753000 | PE150 | 96.99 | 90.98 | 41.89 |
| D31097 | 40156383 | 12046914900 | PE150 | 96.41 | 90.03 | 42.24 |
| D32056 | 40173862 | 12052158600 | PE150 | 98.45 | 94.85 | 42.09 |
| D32069 | 40220000 | 12066000000 | PE150 | 97.96 | 93.49 | 41.98 |
| D33106 | 40120995 | 12036298500 | PE150 | 96.78 | 90.55 | 42.1 |
| D33107 | 40069603 | 12020880900 | PE150 | 98.69 | 95.65 | 42.28 |
| D33111 | 40250454 | 12075136200 | PE150 | 96.44 | 89.88 | 42 |
| D34119 | 40130491 | 12039147300 | PE150 | 97.24 | 92.18 | 42.8 |
| D34125 | 40206811 | 12062043300 | PE150 | 98 | 93.55 | 41.76 |
| D35108 | 39677913 | 11903373900 | PE150 | 96.34 | 89.54 | 41.91 |
| D35109 | 40111276 | 12033382800 | PE150 | 96.68 | 90.7 | 42.26 |
| D35110 | 38315019 | 11494505700 | PE150 | 96.65 | 90.63 | 42.2 |
| D35118 | 40233031 | 12069909300 | PE150 | 97.27 | 91.62 | 41.75 |
| D3513 | 40298010 | 12089403000 | PE150 | 96.66 | 90.18 | 42.3 |
| D36134 | 40225482 | 12067644600 | PE150 | 96.69 | 90.48 | 41.84 |
| D37060 | 39451063 | 11835318900 | PE150 | 95.91 | 88.68 | 42.01 |
| D37061 | 40138215 | 12041464500 | PE150 | 96.74 | 90.32 | 41.67 |
| D37066 | 40057209 | 12017162700 | PE150 | 96.48 | 89.9 | 42.11 |
| D37068 | 40123669 | 12037100700 | PE150 | 95.88 | 89.06 | 42.1 |
| D37070 | 40067805 | 12020341500 | PE150 | 97.64 | 93.29 | 42.65 |
| D38057 | 40159287 | 12047786100 | PE150 | 98.79 | 95.85 | 41.06 |
| D38064 | 40094745 | 12028423500 | PE150 | 96.32 | 89.33 | 41.95 |
| D39054 | 40144064 | 12043219200 | PE150 | 97.42 | 91.96 | 42.11 |
| D39055 | 39991835 | 11997550500 | PE150 | 95.77 | 88.33 | 42.04 |
| D39059 | 40172475 | 12051742500 | PE150 | 96.25 | 89.16 | 41.83 |
| D39060 | 39919834 | 11975950200 | PE150 | 96.39 | 89.67 | 41.54 |
| D39061 | 40117085 | 12035125500 | PE150 | 98.89 | 96.37 | 42.92 |
| D39062 | 40200487 | 12060146100 | PE150 | 96.59 | 90.16 | 42.06 |
| D39063 | 40187938 | 12056381400 | PE150 | 95.74 | 88.7 | 41.58 |
| D39064 | 40185209 | 12055562700 | PE150 | 96.57 | 89.87 | 41.66 |
| D39067 | 40107202 | 12032160600 | PE150 | 98.73 | 95.64 | 42.23 |
| D40068 | 40217174 | 12065152200 | PE150 | 96.24 | 89.28 | 42.14 |
| D40082 | 40238987 | 12071696100 | PE150 | 96.32 | 89.79 | 42.15 |
| D40088 | 40125440 | 12037632000 | PE150 | 97.94 | 93.41 | 42 |
| D40090 | 40260399 | 12078119700 | PE150 | 96.72 | 90.36 | 42.08 |
| D40683 | 40178614 | 12053584200 | PE150 | 96.11 | 89.3 | 42.25 |
| D41054 | 40206111 | 12061833300 | PE150 | 97.88 | 93.21 | 42 |
| D41058 | 40182366 | 12054709800 | PE150 | 96.82 | 90.68 | 42.24 |
| D42103 | 40146705 | 12044011500 | PE150 | 97.69 | 92.64 | 42.03 |
| D42105 | 40078347 | 12023504100 | PE150 | 96.58 | 90.42 | 42.68 |
| D42106 | 36737938 | 11021381400 | PE150 | 97.3 | 91.6 | 42.01 |
| D42108 | 40075822 | 12022746600 | PE150 | 96.43 | 89.48 | 41.68 |
| D43131 | 40177675 | 12053302500 | PE150 | 97.71 | 92.71 | 41.95 |
| D43135 | 40201234 | 12060370200 | PE150 | 97.7 | 92.65 | 41.89 |
| D43136 | 40259487 | 12077846100 | PE150 | 96.49 | 89.66 | 41.51 |
| D44113 | 40240695 | 12072208500 | PE150 | 98.47 | 94.92 | 42.27 |
| D44115 | 40069668 | 12020900400 | PE150 | 96.18 | 89.09 | 41.91 |
| D44117 | 40147115 | 12044134500 | PE150 | 96.2 | 89.25 | 42.01 |
| D44118 | 38595036 | 11578510800 | PE150 | 96.57 | 89.84 | 42.05 |
| D45034 | 40288816 | 12086644800 | PE150 | 96.62 | 90.15 | 41.59 |
| D45036 | 40234624 | 12070387200 | PE150 | 98.29 | 94.48 | 42.48 |
| D46133 | 40091628 | 12027488400 | PE150 | 96.3 | 89.82 | 42.2 |
| D46134 | 40003807 | 12001142100 | PE150 | 96.47 | 90.19 | 42.29 |
| D46135 | 40231980 | 12069594000 | PE150 | 96.15 | 88.87 | 41.36 |
| D46136 | 40083012 | 12024903600 | PE150 | 97.05 | 91.7 | 42.45 |
| D46138 | 40236279 | 12070883700 | PE150 | 96.3 | 89.47 | 41.73 |
| D46144 | 40125662 | 12037698600 | PE150 | 97.95 | 93.92 | 42.88 |
| D47110 | 40187209 | 12056162700 | PE150 | 96.59 | 90.16 | 42.06 |
| D47112 | 40198003 | 12059400900 | PE150 | 96.66 | 90.36 | 41.94 |
| D47115 | 33670010 | 10101003000 | PE150 | 98.49 | 94.97 | 42.46 |
| D47116 | 40047128 | 12014138400 | PE150 | 97.41 | 92.64 | 42.6 |
| D47121 | 40098541 | 12029562300 | PE150 | 96.28 | 89.44 | 42.17 |
| D47124 | 38529129 | 11558738700 | PE150 | 96.09 | 89.34 | 42.16 |
| D47126 | 40167077 | 12050123100 | PE150 | 96.84 | 90.74 | 42.21 |
| D47127 | 40148140 | 12044442000 | PE150 | 96.02 | 89.4 | 41.75 |
| D48131 | 39812488 | 11943746400 | PE150 | 98.83 | 95.98 | 42.41 |
| D48132 | 40050527 | 12015158100 | PE150 | 96.06 | 89.08 | 42.26 |
| D48134 | 39557482 | 11867244600 | PE150 | 95.99 | 89.09 | 42 |
| D48135 | 40169222 | 12050766600 | PE150 | 96.66 | 90.04 | 41.8 |
| D48139 | 40042459 | 12012737700 | PE150 | 95.72 | 88.69 | 41.77 |
| D49131 | 40141617 | 12042485100 | PE150 | 97.49 | 92.09 | 42.04 |
| D51134 | 40129225 | 12038767500 | PE150 | 96.07 | 89.54 | 41.79 |
| D51137 | 40106779 | 12032033700 | PE150 | 96.64 | 90.31 | 41.93 |
| D51142 | 40078416 | 12023524800 | PE150 | 97.42 | 92.07 | 42.08 |
| D52032 | 40189386 | 12056815800 | PE150 | 95.84 | 88.72 | 42.05 |
| D53052 | 37952288 | 11385686400 | PE150 | 96.73 | 90.28 | 42.25 |
| D53054 | 40228844 | 12068653200 | PE150 | 96.63 | 90 | 42.11 |
| D53062 | 40166125 | 12049837500 | PE150 | 98.02 | 93.69 | 41.97 |
| D53063 | 40148216 | 12044464800 | PE150 | 97.06 | 91.23 | 42.23 |
| D54109 | 40069023 | 12020706900 | PE150 | 96.23 | 89.51 | 42.43 |
| D54112 | 40161700 | 12048510000 | PE150 | 98.79 | 95.86 | 42.14 |
| D54114 | 40110101 | 12033030300 | PE150 | 98.38 | 94.62 | 41.98 |
| D54115 | 40226717 | 12068015100 | PE150 | 98.56 | 95.19 | 42.27 |
| D54116 | 39464576 | 11839372800 | PE150 | 96.53 | 89.93 | 42.08 |
| D55053 | 40027501 | 12008250300 | PE150 | 96.7 | 90.36 | 42.23 |
| D55055 | 40132184 | 12039655200 | PE150 | 97.93 | 93.41 | 41.86 |
| D55056 | 40028746 | 12008623800 | PE150 | 96.74 | 90.47 | 42.24 |
| D55057 | 40247633 | 12074289900 | PE150 | 96.19 | 89.21 | 42.06 |
| D55058 | 40114542 | 12034362600 | PE150 | 96.9 | 90.86 | 42.14 |
| D56057 | 37505868 | 11251760400 | PE150 | 96.2 | 89.42 | 42.39 |
| D56058 | 40058200 | 12017460000 | PE150 | 97.93 | 93.45 | 41.79 |
| D56059 | 40048403 | 12014520900 | PE150 | 96.37 | 89.49 | 41.74 |
| D57109 | 40273089 | 12081926700 | PE150 | 95.8 | 88.39 | 42.02 |
| D57110 | 40218884 | 12065665200 | PE150 | 96.5 | 89.81 | 42.09 |
| D57114 | 40121893 | 12036567900 | PE150 | 97.14 | 91.52 | 42.25 |
| D58027 | 40159410 | 12047823000 | PE150 | 95.94 | 89.22 | 42.06 |
| D58030 | 40145477 | 12043643100 | PE150 | 96.47 | 89.89 | 41.67 |
| D58031 | 40212581 | 12063774300 | PE150 | 97.57 | 92.32 | 42.01 |
| D58032 | 40108532 | 12032559600 | PE150 | 96.5 | 89.69 | 42 |
| D58033 | 40143534 | 12043060200 | PE150 | 95.79 | 88.37 | 41.86 |
| D58536 | 40165512 | 12049653600 | PE150 | 98.08 | 93.75 | 41.41 |
| D59106 | 40157607 | 12047282100 | PE150 | 97.97 | 93.57 | 41.89 |
| D59107 | 40062262 | 12018678600 | PE150 | 95.87 | 89.02 | 41.79 |
| D59111 | 39614666 | 11884399800 | PE150 | 95.87 | 88.59 | 42.12 |
| D59112 | 40024579 | 12007373700 | PE150 | 96.45 | 89.53 | 42.08 |
| D59X08 | 40015924 | 12004777200 | PE150 | 96.58 | 90.28 | 42.37 |
| D60105 | 40203609 | 12061082700 | PE150 | 96.53 | 89.81 | 41.84 |
| D60107 | 40151320 | 12045396000 | PE150 | 96.34 | 89.39 | 41.83 |
| D60109 | 40079469 | 12023840700 | PE150 | 98.3 | 94.57 | 42.82 |
| D62137 | 40137304 | 12041191200 | PE150 | 98.13 | 93.93 | 41.95 |
| D62139 | 40211719 | 12063515700 | PE150 | 96.32 | 89.36 | 41.73 |
| D62140 | 40218658 | 12065597400 | PE150 | 96.28 | 89.42 | 41.88 |
| D63126 | 36385979 | 10915793700 | PE150 | 98.54 | 95.13 | 42.2 |
| D63128 | 40094669 | 12028400700 | PE150 | 96.78 | 90.5 | 42.15 |
| D64107 | 40221484 | 12066445200 | PE150 | 98.93 | 96.37 | 42.8 |
| D64113 | 40149568 | 12044870400 | PE150 | 95.49 | 88.12 | 41.91 |
| D65013 | 40152459 | 12045737700 | PE150 | 98.12 | 93.91 | 41.96 |
| D65014 | 40142832 | 12042849600 | PE150 | 97.07 | 91.27 | 42.29 |
| D65015 | 40096735 | 12029020500 | PE150 | 96.83 | 90.74 | 42.27 |
| D65018 | 40122639 | 12036791700 | PE150 | 98.61 | 95.2 | 42.06 |
| D65019 | 40226239 | 12067871700 | PE150 | 96.43 | 90.05 | 42.13 |
| D65023 | 40163393 | 12049017900 | PE150 | 95.98 | 88.64 | 41.79 |
| D65024 | 40131731 | 12039519300 | PE150 | 96.25 | 89.58 | 42.35 |
| D65026 | 40157482 | 12047244600 | PE150 | 96.25 | 89.28 | 41.67 |
| D66051 | 40152930 | 12045879000 | PE150 | 96.01 | 89.11 | 42.04 |
| D66060 | 40072941 | 12021882300 | PE150 | 97.6 | 92.48 | 41.92 |
| D67133 | 40172429 | 12051728700 | PE150 | 96.3 | 89.65 | 42.29 |
| D67135 | 40086196 | 12025858800 | PE150 | 96.45 | 90.05 | 42.14 |
| D67136 | 40153943 | 12046182900 | PE150 | 96.56 | 90.16 | 41.91 |
| D67139 | 38100301 | 11430090300 | PE150 | 98.15 | 94.1 | 42.14 |
| D6740 | 40173916 | 12052174800 | PE150 | 96.53 | 90.03 | 40.88 |
| D68111 | 40095826 | 12028747800 | PE150 | 96.41 | 89.61 | 42.04 |
| D68125 | 40132369 | 12039710700 | PE150 | 97.84 | 93 | 41.58 |
| D68131 | 40084857 | 12025457100 | PE150 | 98.43 | 94.81 | 42.32 |
| D68132 | 40136790 | 12041037000 | PE150 | 97.51 | 92.21 | 42.21 |
| D68134 | 40271569 | 12081470700 | PE150 | 96.75 | 90.33 | 42.01 |
| D68136 | 40130228 | 12039068400 | PE150 | 97.94 | 93.31 | 41.75 |
| D68137 | 40247408 | 12074222400 | PE150 | 97.73 | 92.77 | 41.87 |
| D68139 | 40122745 | 12036823500 | PE150 | 98.15 | 93.99 | 41.87 |
| D68143 | 40005227 | 12001568100 | PE150 | 97.79 | 93.03 | 41.84 |
| D68144 | 40211697 | 12063509100 | PE150 | 97.91 | 93.35 | 42.17 |
| D69009 | 40192602 | 12057780600 | PE150 | 97.56 | 92.32 | 42.1 |
| D69011 | 40171089 | 12051326700 | PE150 | 96.23 | 89.28 | 41.93 |
| D69012 | 40136771 | 12041031300 | PE150 | 97.19 | 91.66 | 42.04 |
| D69014 | 40176594 | 12052978200 | PE150 | 95.89 | 88.85 | 41.99 |
| D69015 | 40100193 | 12030057900 | PE150 | 96.27 | 89.61 | 42.53 |
| D69016 | 40156112 | 12046833600 | PE150 | 97.98 | 93.48 | 41.97 |
| D69017 | 40175608 | 12052682400 | PE150 | 96.56 | 90.07 | 41.48 |
| D71058 | 40184142 | 12055242600 | PE150 | 97.38 | 91.81 | 42.11 |
| D71060 | 40216358 | 12064907400 | PE150 | 96.66 | 90.42 | 42.22 |
| D71064 | 40169059 | 12050717700 | PE150 | 96.02 | 88.98 | 42.34 |
| D71065 | 40013179 | 12003953700 | PE150 | 98.01 | 93.53 | 41.69 |
| D71066 | 40203368 | 12061010400 | PE150 | 96.2 | 89.54 | 42.1 |
| D71067 | 40121673 | 12036501900 | PE150 | 98.07 | 93.76 | 41.73 |
| D71069 | 40036007 | 12010802100 | PE150 | 95.53 | 87.65 | 41.84 |
| D71070 | 40200836 | 12060250800 | PE150 | 98.62 | 95.4 | 42.13 |
| D72127 | 38329027 | 11498708100 | PE150 | 96.98 | 91.06 | 42.4 |
| D72130 | 40138778 | 12041633400 | PE150 | 98.07 | 93.75 | 41.77 |
| D72132 | 40091078 | 12027323400 | PE150 | 96.65 | 90.07 | 41.62 |
| D72137 | 39960674 | 11988202200 | PE150 | 96.54 | 89.76 | 42.12 |
| D72140 | 40049152 | 12014745600 | PE150 | 96.87 | 90.73 | 41.79 |
| D72141 | 40197954 | 12059386200 | PE150 | 98.13 | 93.93 | 41.91 |
| D73018 | 40187331 | 12056199300 | PE150 | 96.19 | 89.49 | 42.12 |
| D73020 | 40112891 | 12033867300 | PE150 | 96.18 | 89.37 | 42.35 |
| D73023 | 40122318 | 12036695400 | PE150 | 96.15 | 89.49 | 42.14 |
| D74014 | 40090421 | 12027126300 | PE150 | 96.59 | 89.97 | 42.17 |
| D74015 | 40159469 | 12047840700 | PE150 | 98.47 | 94.92 | 42.19 |
| D74016 | 40133012 | 12039903600 | PE150 | 98.48 | 94.94 | 42.17 |
| D74020 | 40161435 | 12048430500 | PE150 | 97.93 | 93.24 | 41.72 |
| D74021 | 40246595 | 12073978500 | PE150 | 95.76 | 88.52 | 42.19 |
| D74022 | 40095358 | 12028607400 | PE150 | 98.56 | 95.2 | 42.46 |
| D74023 | 40059248 | 12017774400 | PE150 | 96.03 | 89.42 | 41.69 |
| D74027 | 40167731 | 12050319300 | PE150 | 96.28 | 89.78 | 42.15 |
| D75029 | 40144789 | 12043436700 | PE150 | 97.33 | 92.02 | 42.61 |
| D75104 | 40136873 | 12041061900 | PE150 | 97.96 | 93.49 | 41.65 |
| D75110 | 40181990 | 12054597000 | PE150 | 98.07 | 93.78 | 41.85 |
| D75112 | 40254940 | 12076482000 | PE150 | 96.78 | 90.57 | 42.22 |
| D76056 | 40128940 | 12038682000 | PE150 | 97.63 | 92.56 | 42.23 |
| D76057 | 40149803 | 12044940900 | PE150 | 96.5 | 90.23 | 42.13 |
| D76059 | 40023672 | 12007101600 | PE150 | 97.77 | 93.6 | 43.1 |
| D76064 | 40163766 | 12049129800 | PE150 | 96.56 | 90.12 | 42.02 |
| D77026 | 40104610 | 12031383000 | PE150 | 96.71 | 90.26 | 41.63 |
| D77027 | 40096018 | 12028805400 | PE150 | 97.42 | 91.95 | 42.09 |
| D77028 | 40170330 | 12051099000 | PE150 | 96.58 | 89.94 | 41.99 |
| D77034 | 39472192 | 11841657600 | PE150 | 96.51 | 89.64 | 42.12 |
| D77038 | 40053629 | 12016088700 | PE150 | 96.94 | 91.16 | 42.62 |
| D78057 | 40178096 | 12053428800 | PE150 | 97.89 | 93.31 | 41.92 |
| D78059 | 40191135 | 12057340500 | PE150 | 96.3 | 89.49 | 41.77 |
| D78060 | 40185916 | 12055774800 | PE150 | 97.91 | 93.24 | 41.84 |
| D79105 | 40178525 | 12053557500 | PE150 | 95.91 | 88.66 | 41.98 |
| D79107 | 40201169 | 12060350700 | PE150 | 96.36 | 89.92 | 42.15 |
| D79111 | 40077744 | 12023323200 | PE150 | 98.14 | 93.93 | 41.43 |
| D80108 | 40101146 | 12030343800 | PE150 | 96.67 | 90.36 | 42.19 |
| D80110 | 40070902 | 12021270600 | PE150 | 96.25 | 89.32 | 41.84 |
| D80114 | 40126943 | 12038082900 | PE150 | 96.39 | 89.56 | 41.95 |
| D80116 | 40135030 | 12040509000 | PE150 | 96.56 | 90.42 | 42.09 |
| D80117 | 40076901 | 12023070300 | PE150 | 98.08 | 93.73 | 41.62 |
| D80119 | 40148437 | 12044531100 | PE150 | 98.48 | 94.97 | 42.36 |
| D83052 | 40198357 | 12059507100 | PE150 | 98.69 | 95.5 | 42.16 |
| D84074 | 40178829 | 12053648700 | PE150 | 96.27 | 89.7 | 41.99 |
| D85060 | 40076552 | 12022965600 | PE150 | 98.6 | 95.48 | 42.72 |
| X57112 | 40089956 | 12026986800 | PE150 | 96.66 | 90.62 | 42.05 |
