# Supplementary material for: Genome-wide association study reveals putative genetic determinants of longitudinal immune responses to avian influenza and Newcastle disease in chickens
Source: Genet Sel Evol. 2026 Aug 11;58:56. doi: 10.1186/s12711-026-01077-2 (PMC13501648; doi:10.1186/s12711-026-01077-2)
Supplement: Supplementary file 5 [file 12711_2026_1077_MOESM6_ESM.docx]

**Additional files**

**Additional file 1: Figure S1 (a-b)**

File format: PDF file

Title**:** Normality assessment of antibody response phenotypes. (a) Histogram of avian influenza virus (AIV) antibody response phenotypes. (b) Histogram of Newcastle disease virus (NDV) antibody response phenotypes. Shapiro-Wilk tests indicated that both traits were normally distributed (*P* = 0.452 and *P* = 0.353 for AIV and NDV, respectively).

**Additional file 1: Figure S2**

File format: PDF file

Title: Manhattan plots (left panels) and quantile-quantile (Q-Q) plots (right panels) for avian influenza virus (AIV) H5, H7, and H9 antibody responses and Newcastle disease virus (NDV) antibody response at P0. The red horizontal line indicates the genome-wide significance threshold, and the blue dashed line indicates the suggestive significance threshold. The close agreement between observed and expected *P*-value distributions in the Q-Q plots indicates that population structure and relatedness were adequately controlled in the association analyses.

**Additional file 2: Tables S1-S3**

File format: Docx file

Title: Supplementary tables containing phenotypic and genetic correlations and sequencing summary statistics. Table S1. Phenotypic and genetic correlations among all avian influenza virus (AIV) subtype antibody response traits measured in 572 chickens. Table S2. Phenotypic and genetic correlations among Newcastle disease virus (NDV) antibody response traits measured in 572 chickens. Table S3. Summary statistics of sequencing data for 587 individual samples, including the numbers of clean reads and clean bases obtained after quality control.

**Additional file 3: Figure S3 (a-b)**

File format: PDF file

Title: Linkage disequilibrium analysis of the significant genomic region. (a) Regional association plot highlighting the significant SNP cluster within the candidate genomic region. (b) Linkage disequilibrium (LD) heatmap generated from SNPs within the candidate region. LD analysis identified five haplotype blocks spanning the associated interval.

**Additional file 4: Tables S4-S5**

File format: xlsx file

Title**:** Candidate genes associated with antibody responses to avian influenza virus (AIV) and Newcastle disease virus (NDV). Table S4. Candidate genes associated with AIV antibody response traits identified through GWAS. Table S5. Candidate genes associated with NDV antibody response traits identified through GWAS.

**Additional file 5: Table S6**

File format: xlsx file

Title: Gene Ontology enrichment analysis of candidate genes identified from GWAS. The table includes Gene Ontology (GO) categories, enriched GO terms, gene counts, enrichment *P*-values, and the corresponding candidate genes associated with each enriched term.
